# Age-dependent regulation of ELP1 exon 20 splicing in Familial Dysautonomia by RNA Polymerase II kinetics and chromatin structure

**DOI:** 10.1371/journal.pone.0298965

**Published:** 2024-06-03

**Authors:** Federico Riccardi, Giulia Romano, Danilo Licastro, Franco Pagani

**Affiliations:** 1 Human Molecular Genetics, International Centre for Genetic Engineering and Biotechnology, Padriciano, Trieste, Italy; 2 Laboratorio di Genomica ed Epigenomica, AREA Science Park, Padriciano, Trieste, Italy; Saint Louis University School of Medicine, UNITED STATES

## Abstract

Familial Dysautonomia (FD) is a rare disease caused by ELP1 exon 20 skipping. Here we clarify the role of RNA Polymerase II (RNAPII) and chromatin on this splicing event. A slow RNAPII mutant and chromatin-modifying chemicals that reduce the rate of RNAPII elongation induce exon skipping whereas chemicals that create a more relaxed chromatin exon inclusion. In the brain of a mouse transgenic for the human FD-ELP1 we observed on this gene an age-dependent decrease in the RNAPII density profile that was most pronounced on the alternative exon, a robust increase in the repressive marks H3K27me3 and H3K9me3 and a decrease of H3K27Ac, together with a progressive reduction in ELP1 exon 20 inclusion level. In HEK 293T cells, selective drug-induced demethylation of H3K27 increased RNAPII elongation on ELP1 and SMN2, promoted the inclusion of the corresponding alternative exons, and, by RNA-sequencing analysis, induced changes in several alternative splicing events. These data suggest a co-transcriptional model of splicing regulation in which age-dependent changes in H3K27me3/Ac modify the rate of RNAPII elongation and affect processing of ELP1 alternative exon 20.

## Introduction

Alternative splicing (AS) is the dynamic process by which different sets of exonic sequences from a single pre-mRNA molecule are spliced together to obtain different mRNA isoforms, allowing an extraordinary increase in transcriptome and proteome diversity [1, 2]. AS and in particular its most common event, exon skipping, is determined by a coordinated interplay involving different regulators that act co-transcriptionally [3]. Alternative spliced exons are regulated by binding on nascent pre-mRNA of activator and repressor RNA binding proteins (RBPs) to enhancers and silencers, respectively [4–6], and also by the rate of RNA Polymerase II (RNAPII) transcription elongation and chromatin organization [7, 8]. Numerous studies indicate that RNAPII activity can modulate AS through a kinetic coupling mechanism. This model assumes that the speed of elongating RNAPII influences the timing of presentation of splice sites and regulatory sequences gradually emerging from the transcribing pre-mRNA, promoting either skipping or inclusion of the alternative spliced exon [9–15]. Approximately 20% of alternative splicing events are sensitive to RNAPII elongation: depending on the effect that RNAPII rate has on splicing, alternative exons are mainly classified into type I and type II, where slow elongation promotes respectively the inclusion or skipping of the exon [16–18]. RNAPII elongation can be also regulated by chromatin. Several histone modifications have been shown to affect AS by altering RNAPII kinetics through the creation of a more compact or relaxed chromatin that creates or releases a roadblock to transcript elongation, respectively [15, 19–23]. Recently, an epigenetic therapy strategy based on HDAC inhibitors to modulate chromatin marks was used to improve in an animal model with Spinal Muscular Atrophy (SMA) the inclusion of the type II SMN2 exon 7, increasing the therapeutic efficacy of an antisense oligonucleotide (ASO)-splicing therapy [15]. Chromatin marks can also affect alternative splicing through direct recruitment of splicing factors [21, 24–28]. AS is temporally regulated and contributes to tissue-identity acquisition and maintenance, cell differentiation and organ development [2]. Some splicing factors, mainly with tissue-specific expression, have been shown to contribute to this temporal regulation [29–34]. Chromatin remodelling is also a fundamental event during development and aging [35–38] but its potential impact on AS and in particular on aberrant splicing events is not clear, leaving the temporal contribution of chromatin organization and RNAPII transcription elongation on AS largely unexplored. We focused here on Familial Dysautonomia (FD), a rare recessive autosomal disorder characterized by progressive degeneration of the sensory and autonomic nervous system [39, 40]. Over 99.5% of affected individuals are homozygous for a point mutation in ELP1 (Elongator Acetyltransferase Complex Subunit 1) intron 20, where T is switched with C in position +6 [41–43] leading to exon 20 skipping. This aberrant splicing (here referred to as the FD-ELP1 defect) has a tissue variability with highest levels of exon 20 skipping in neuronal tissue [44]. Therapeutic strategies to rescue the FD splicing defect include antisense oligonucleotides (ASO) [45], modified U1s [46, 47] and chemicals [48–51]. Using a slow RNAPII kinetic mutant and chemicals that either affects RNAPII elongation or alter global chromatin status we demonstrate that the FD-ELP1 exon 20 splicing event follows the kinetic coupling model of co-transcriptional splicing and belongs to type II alternative exons. Investigating the temporal changes of ELP1 exon 20 defective splicing in FD transgenic and asymptomatic mouse model, we show that this event is modulated in an age-dependent and tissue-specific manner. In brain, we show that the temporal decrease in ELP1 exon 20 inclusion is associated to the acquisition of repressive mark H3K27me3 and to the loss of active mark H3K27Ac, with a concomitant reduction in RNAPII density profile along the entire ELP1 gene. In a cellular model, drug-induced depletion of H3K27me3 modulates splicing on a global basis. On ELP1 and SMN2 genes, H3K27me3 reduction increases RNAPII elongation and promotes the inclusion of corresponding defective exons. Our results suggest that temporal changes in chromatin marks modulate splicing of FD-ELP1.

## Material and methods

### Animal model and mouse organs collection

All animals were housed and handled in a controlled environment (22°C with a 12 hours light /dark photoperiod) and provided with free access to food and water in conformity with the International Centre for Genetic Engineering and Biotechnology institutional guidelines and in compliance with national and international laws and policies (EU Directive 2010/63/EU) upon approval by the Italian Ministry of Health. The transgenic FD mouse model (*Ikbkap*^*+/+*^*; TgFD9*^*+/+*^*)* has been previously described [43]. After euthanasia, which was performed using inhalant anaesthetic overdose (isofluorane) followed by decapitation, organs of interest were collected in Safe-Lock tubes (Eppendorf) at three time points: post-natal days (P) 10, 90 and 365. The dissection was performed in PBS buffer on an ice-cold plate and the extracted samples were immediately frozen in liquid nitrogen and stored at -80°C. RNA, protein and chromatin analyses were performed as described in the respective paragraphs.

### Cell culture, transfections and chemicals addition

HEK 293T cells were grown in Dulbecco’s Modified Eagle Medium with Glutamax I (Gibco, Life Technologies) supplemented with 10% fetal calf serum (FBS) (Gibco, Life Technologies) and antibiotic antimycotic (Sigma) according to manufacturer’s instruction. Cells were kept in humidified incubator at 37°C and 5% of CO_2_. Cells were plated at a density of ~3x10^5^ cells in 6-well plates 24 hours before co-transfection. Co-transfection experiments were carried out using Effectene transfection reagent (Qiagen) according to manufacturer’s instruction using 1.5 ug of pTB-ELP1 mutant minigene and 1.5 ug or 3 ug of α-amanitin-resistant variants of the large subunit of human RNA Polymerase II wild type (WTres; pAT7Rpb1αAmr vector) and the slow R749H mutant (pAT7Rpb1αAmrR749H vector). At the time of co-transfection, 2.5 ug/ul or 5 ug/ul of α-amanitin were added to cells to block endogenous RNA Polymerase II transcription and after 24 hours, cells were harvested and subjected to further analysis. For experiments with chemicals in HEK 293T cells, 0.5 ug of pTB-ELP1 mutant minigene were transfected and 4 mM Valproic acid (VPA) (Sigma), 1 ug/mL Trichostatin A (TSA) (Sigma), 100 uM EED226 (MedChemExpress), 12 uM Camptothecin (CPT) (Sigma) and 75 uM 5,6-Dichloro-1-β-D-ribofuranosylbenzimidazole (DRB) (Sigma) were added to cells at the time of transfection. Cells were harvested after 24 h for and subjected to further analysis. Human FD and WT fibroblasts were purchased from the Coriell Institute (GM04589 and GM02674B, respectively) and maintained as suggested by the supplier. For experiments with chemicals in FD and WT primary cells, 8 mM VPA, 1 ug/mL TSA, 100 uM EED226, 12 uM CPT and 20 uM DRB were added to cells plated in 12-well plates and after 24 hours fibroblasts were harvested and subjected to further analysis. In HEK 293T cells and fibroblasts experiments, 0.1% DMSO (Sigma) (for VPA, TSA, EED226, CPT and DRB) was used as control.

### RNA isolation and splicing analysis

Total RNA was extracted with TRIzol (ThermoFisher), treated with DNase (Invitrogen) and 150 ng–1 ug of RNA were reverse transcribed using Superscript Vilo MasterMix (ThermoFisher) all according to manufacturer’s instruction. *In vivo* and *ex vivo* human ELP1 exon 20 splicing and total ELP1 mRNA analyses were performed by end-point RT-PCR (ePCR) and quantitative RT-PCR (qRT-PCR) using iQ SYBR Green in a CFX96 Real-Time PCR system (Bio-Rad Laboratories) as previously described [46, 47]. GAPDH was used as housekeeping gene. *In vitro* minigene splicing patterns of ELP1 exon 20, HBA exon 2, SMN2 exon 7, CFTR exon 9 (wt and mutated), FN1 exon 33, FN1 exon 25 and FIX exon 5Δ6 and endogenous SMN2 exon 7, ELP1 exon 10 and exon 20 were analyzed by ePCR with specific primers (S1 Table).

### DRB-RNA Polymerase II measurement

RNA Polymerase II elongation analysis was performed adapting the method developed by [14, 52] with some exceptions. HEK 293T cells were seeded at a density of ~1x10^5^ cells in P35 plates and after 24 hours were treated with 100 uM DRB for 5 hours to fully block ELP1 transcription. For CPT-treated cells, CPT 1 uM was added 30 minutes before the DRB-wash whereas for EED226-treated cells, EED226 100 uM was added 24 hours before DRB treatment and both drugs, respectively, remained until cell harvest. Total RNA was extracted as mentioned above, treated with DNase and RT-PCR was performed. Quantification of pre-mRNAs was performed by qPCR using iQ SYBR Green in a CFX96 Real-Time PCR system (Bio-Rad Laboratories) with amplicons spanning the intron-exon junction and results were expressed in relation to pre-mRNA value of cells never treated with DRB. U6 snRNA, being transcribed by RNA Polymerase III, was used as housekeeping gene in this analysis. Primers are found in S1 Table.

### Protein isolation and western blot analysis

Previously collected mouse brains were transferred in ice-cold RIPA buffer (Sigma) containing Protease Inhibitor Cocktail (Roche), completely homogenized using ceramic beads MagNA Lyser (Roche Diagnostic) and debris were discarded after centrifugation. HEK 293T cells and fibroblasts lysates were obtained by lysis with ice-cold RIPA buffer (Sigma) and Protease Inhibitor Cocktail (Roche). For both mouse brains and cells, protein samples were sonicated (Bandelin Sonopuls) and protein concentration was measured with protein assay dye reagent (Bio-Rad Laboratories). A total of 20 ug of protein was separated on NuPAGE 4–12% Bis-Tris precast gels (ThermoFisher) and transferred to 0.2 um nitrocellulose membranes (Amersham). Membranes were blocked for 1 hour at room temperature with 5% non-fat milk dissolved in PBS-T/TBS-T (Tween20) 0.1% and after probing with suitable primary and HRP-conjugated secondary antibodies, chemiluminescence signals were captured with UVItec (CAMBRIDGE Alliance). Western blot analyses were performed with anti-GAPDH (1:5000; ab8245, Abcam), anti-histone H3 (1:15000; ab1791, Abcam), anti-H3K27me3 (1:1000; ab6002, Abcam) and anti-H3K27Ac (1:1000; ab4729, Abcam). Anti-mouse (1:2000; Dako) and anti-rabbit (1:2000; Dako) were used as secondary antibodies.

### Chromatin immunoprecipitation

Chromatin immunoprecipitation of mouse brains at P10, P90 and P365 was performed using EpiQuik Tissue Chromatin Immunoprecipitation kit (Epigentek, catalog # P2003) according to manufacturer’s instructions with minor modifications. Briefly, 150 mg of frozen tissue were cut into small pieces with a blade in ice-cold PBS 1X, sucked in 18 G needle syringe, cross-linked with 1% formaldehyde for 10 min at room temperature and quenched with 150 ul of PBS 1X-Glycine 1.25M for 10 min at room temperature. Then, lysates were centrifuge for 5 minutes at room temperature at 800 RPM, the supernatant was discarded and an additional round of PBS 1X-Glycine was added to the lysate, followed by a wash with ice-cold PBS 1X. Samples were homogenized using a Douncer homogenizer and centrifuged to pellet nuclei at 5000 RPM for 5 minutes at +4°C. After homogenization, lysis buffer was added to nuclei and they were sucked in 18 G needle syringe. Chromatin was prepared and sonicated using a water bath Bioruptor (Diagenode) (30” ON/30” OFF, High power, 3 x 10 cycles) to a size range of 200–1000 bp. To pre-cleared cell debris, sonicated chromatin was centrifuged at 14.000 RPM at +4°C for 10 minutes. Chromatin was diluted and ChIP performed according to manufacturer’s instructions using antibodies against Rpb1 NTD (2 ug; D8L4Y, Cell Signalling Technology), histone H3 (3.5 ug; ab1791, Abcam), H3K9me3 (3.5 ug; ab8898, Abcam), H3K27me3 (3.5 ug; ab6002, Abcam) and H3K27Ac (3.5 ug; ab4729, Abcam). IgG1 (2 ug; G3A1, Cell Signalling Technology) was used as negative control in the immunoprecipitation. Immunoprecipitated DNA was purified by phenol-chloroform extraction and in parallel 5 ul (5%) were taken to be used as input in the quantification analysis. qPCRs were performed using iQ SYBR Green in a CFX96 Real-Time PCR system (Bio-Rad Laboratories) and data were plotted as mean ± s.e.m. Primers are found in S1 Table.

### RNA sequencing and data analysis

Messenger RNA sequencing (RNA-seq) was performed by Area Science Park Sequencing facility. HEK 293T cells treated with EED226 100 uM for 48h (n = 3) and control cells (n = 3) were purified with TRIazol (Ambion) and quality of total RNA was assessed using Agilent 2100 nano bioanalyzer microfluidic chips and a Nanodrop UV spectrophotometer (ThermoFisher). Only RNA with a RIN value of 9.0 or higher and a 28s/18s ratio 1.8 was taken forward for sample preparation. The size distribution of HEK 293T cells was estimated by electrophoresis on Agilent high-sensitivity bioanalyzer microfluidic chips and yield was quantified using the KAPA library quantification kit (KK4824, Kapa Biosystems). Library was pooled at equimolar concentrations and diluted before loading onto the flow cell of a NovaSeq 6000 (Illumina) for both clustering and sequencing. Amplified clusters in the flow cell were then sequenced with 150-base paired-end reads using the NovaSeq 6000SP Reagent Kit v1 (300 cycle) (Illumina Inc.). Real-time image analysis and base calling were performed on a NovaSeq 6000 instrument using the recommended sequencing control software. Illumina standard software was used for de-multiplexing and producing FASTQ sequence files. FASTQ raw sequence files were subsequently quality checked with FASTQC software (v.0.11.3 http://www.bioinformatics.bbsrc.ac.uk/projects/fastqc) and sequences including adaptor dimers, mitochondrial, or ribosomal sequences were discarded. The resulting set of trimmed reads from HEK 293T cells was then mapped onto human GRCh38/hg38 using the Spliced Transcripts Alignment to a Reference (STAR) algorithm [53]. Differential gene expression analysis from HEK 293T cells was performed by the Bioconductor package DESeq2 (v.1.32) using default parameters [54]. To detect outlier data after normalization, we used R packages, and before testing differential gene expression, we dropped all genes with low normalized mean counts to improve testing power while maintaining type I error rates. Estimated false discovery rate (FDR) values for each gene were adjusted using the Benjamini-Hochberg method. Prior to analysis, genes without a poly-A tail were discarded. Features with baseMean ≥ 50 counts, padj ≤ 0,5 and absolute logarithmic base 2-fold change (log_2_FC) ≤ -1 or ≥ 1 were considered having a significant altered expression. For genome-wide splicing analysis, BAM files produced from STAR mapping were input into rMATS [55], using Human GRCh38/hg38 annotation. For detection of alternative splicing (AS) patterns, human annotation was generated containing all consecutive spliced and unspliced exon-intron-exon triads from hg38 (Gencode v29). Five basic types of AS were analyzed: skipped exons (SE), retained introns (RI), mutually exclusive exons (MXE), alternative 5’ splice sites (A5SS) and alternative 3’ splice sites (A3SS). Read coverage was based on actual reads as used in Irimia et al. [56]; SE, RI, and MXE types with an actual read mapping to all exclusion splice junction ≥ 20 were considered whereas for A5SS and A3SS types ≥ 40 actual reads mapping to the sum of all splice junctions involved in the specific event were considered. Estimated FDR values for each gene were adjusted using the Benjamini-Hochberg method. The threshold parameters were set at FDR value ≤ 0,5 and absolute inclusion level difference ≤ -0,05 or ≥ 0,05.

### Pathway analysis by Ingenuity Pathway Analysis

The list of significant differentially expressed genes in EED226-treated HEK 293T cells was uploaded into the IPA software (Qiagen). The ‘‘core analysis” function included in the software was used to interpret the differentially expressed data, which included biological processes, canonical pathways, and gene networks. Each gene identifier was mapped to its corresponding gene object in the Ingenuity Pathway Knowledge Base (IPKB).

### Statistical analysis

A statistical analysis was performed on the investigated groups of data using Prism software version 8.0 (GraphPad Software, La Jolla, CA). The exact sample size and/or biological replicates are reported in figure legends. In all *in vitro* and *ex vivo* experiments, Two tailed Student’s t-test (also known as two-samples t-test) was used (p>0.05: not significant (ns); p<0.05*; p<0.01**; p<0.001***) while in all *in vivo* experiments, One-Way ANOVA test was used applying Bonferroni correction (p>0.05: not significant (ns); p<0.05*; p<0.01**; p<0.001***). To perform these tests, all the *in vitro* and *ex vivo* experiments were repeated three times in triplicate and all the *in vivo* experiments were repeated two times in triplicate but ChIP analysis, in which for each time-point a pool of 3 mice was considered and two biological replicates were performed. Data are expressed as mean ± SD or s.e.m. as indicated in the figure legend.

## Results

### FD-ELP1 exon 20 splicing is co-transcriptionally regulated by RNA Polymerase II elongation rate and HDAC inhibitors

**Fig 1:**
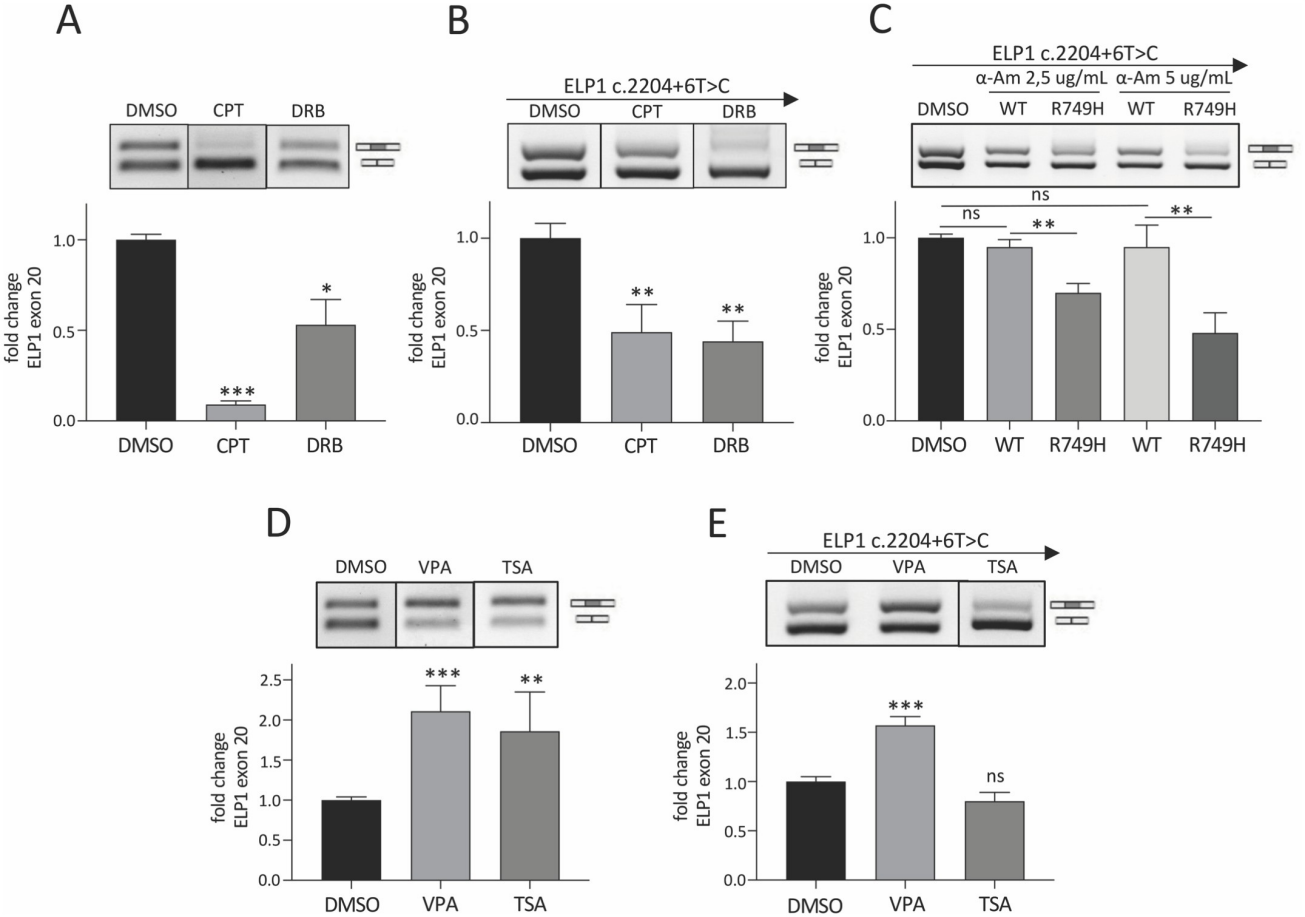
RNA Polymerase II elongation rate and chromatin structure affect FD-ELP1 exon 20 splicing. (**A**) FD-patients’ fibroblasts were treated with 0.1% DMSO or CPT 12 uM or DRB 20 uM for 24 h. DMSO-treated cells are set to 1. (**B**) HEK 293T cells were transfected with ELP1 mutant minigene (c.2204+6T>C) and treated with 0.1% DMSO or CPT 12 uM or DRB 75 uM for 24 h. DMSO-treated cells are set to 1. (**C**) HEK 293T cells were co-transfected with ELP1 mutant minigene and the expression vectors for WTres^Pol II^ and R749H slow mutant RNAPII followed by the addition of 2.5 ug/mL or 5 ug/mL of α-amanitin for 24h. Cells transfected only with ELP1 mutant minigene and treated with 0.1% DMSO are set to 1. (**D**) FD-patients’ fibroblasts were treated with 0.1% DMSO or VPA 8 mM or TSA 1 ug/mL for 24 h. DMSO-treated cells are set to 1. (**E**) HEK 293T cells were transfected with ELP1 mutant minigene and treated with 0.1% DMSO or VPA 4 mM or TSA 1 ug/mL for 24 h. DMSO-treated cells are set to 1. In panels (A) and (D) the upper band of 202 bp corresponds to transcripts including exon 20; the lower band of 128 bp to exon 20 skipping, whereas in panels (B), (C) and (E) the upper band of 349 bp corresponds to transcripts including exon 20; the lower band of 275 bp to exon 20 skipping. In all panels, the intensity of the bands was measured with ImageJ software and histogram below gel displays the percentage (%) of ELP1 exon 20 inclusion expressed as fold change. Dividing lines indicate cropping and annealing of the same agarose gel or experiment. Data are expressed as mean + S.D. of n = 3 experiments in triplicate. Statistical analysis was performed using Student t-test (ns: not significant; *p<0.05; **p<0.01; *** p<0.001).

To understand the role of RNA Polymerase II (RNAPII) kinetics on FD-ELP1 exon 20 splicing, we evaluated the effect of Camptothecin (CPT) and DRB, two chemicals that inhibit the rate of RNAPII elongation [57–59]. CTP blocks the Topoisomerase 1 (TOP1) at the replication bubble and DRB the kinase activity of the CDK9 subunit of the Positive transcription elongation factor (P-TEFb). The effect of these drugs was tested on ELP1 exon 20 splicing in FD patients’ fibroblasts and in minigenes in transient transfection experiments. These drugs induced a significant decrease in the percentage of exon 20 inclusion in both FD fibroblasts and minigene experiments (Fig 1A and 1B). We observed a ~10- and ~2-folds decrease in the ELP1 exon 20 splicing in the FD fibroblasts, and a ~1.5- to ~2.5-fold decrease in minigene assay after CPT and DRB treatment, respectively. The drugs did not affect either α-globin (HBA) exon 2 splicing in ELP1 minigene, ELP1 exon 10 and exon 20 in HEK 293T cells or exon 20 in WT fibroblasts (S1A–S1D Fig). Next, to better elucidate the effect of RNAPII rate on alternative splicing of ELP1 exon 20 we evaluated a RNAPII kinetic mutant, R749H, which has significantly reduced (~2–3.5- folds) RNAPII speed [17, 18]. In this experiment, we co-transfected HEK 293T cells with the FD ELP1 minigene along with plasmid that encode this α-amanitin resistant RNAPII kinetic mutant. Cells were treated with α-amanitin to block endogenous RNAPII transcription, followed by the evaluation of exon 20 splicing pattern. Notably, we found an α-amanitin dose-dependent decrease of exon 20 inclusion, from ~1.6- to ~2.5-folds, when transcription was carried out by the slow RNAPII (Fig 1C). Constitutive HBA exon 2 and ELP1 exon 10 were not affected by the RNAPII mutant (S1E and S1F Fig). These results indicate that ELP1 exon 20 inclusion, as previously reported for two other disease-associated exons, CFTR exon 9 and SMN2 exon 7 [14, 15], is negatively regulated by the speed of RNAPII. Since RNAPII elongation can be modulated by chromatin structure, we evaluated the effect of two global histone deacetylase (HDAC) inhibitors, Valproic acid (VPA) and Trichostatin A (TSA) on ELP1 exon 20 splicing in FD fibroblasts and in minigene experiments. These drugs, reducing the deacetylation of several histones, promote RNAPII elongation [14, 15]. In FD fibroblasts, both drugs had a positive effect on ELP1 splicing, with a ~2-folds increase in exon 20 inclusion (Fig 1D and 1E). In minigene experiments, VPA increased exon 20 inclusion by ~1.5-fold, whereas TSA had no effect (Fig 1D and 1E). These HDAC inhibitors had no effect on constitutive control exons (S1G–S1J Fig). The more pronounced effect of CPT on ELP1 exon 20 splicing in FD fibroblasts compared to the one achieved in HEK 293T cells, and the lack of effect of TSA in the same cells, could be related to the different composition of the chromatin between different cell types (primary versus immortalized cells) and to experimental conditions (endogenous versus minigene context). Thus, chemical compounds that create a more relaxed chromatin structure and in turn promote RNAPII elongation have a positive effect on the inclusion of ELP1 FD exon 20. Collectively, these results indicate that global changes in the chromatin composition regulate ELP1 exon 20 alternative splicing through RNAPII elongation.

### Age-dependent changes of splicing and RNA Polymerase II density profile in hELP1 gene in brain of FD transgenic mouse

**Fig 2:**
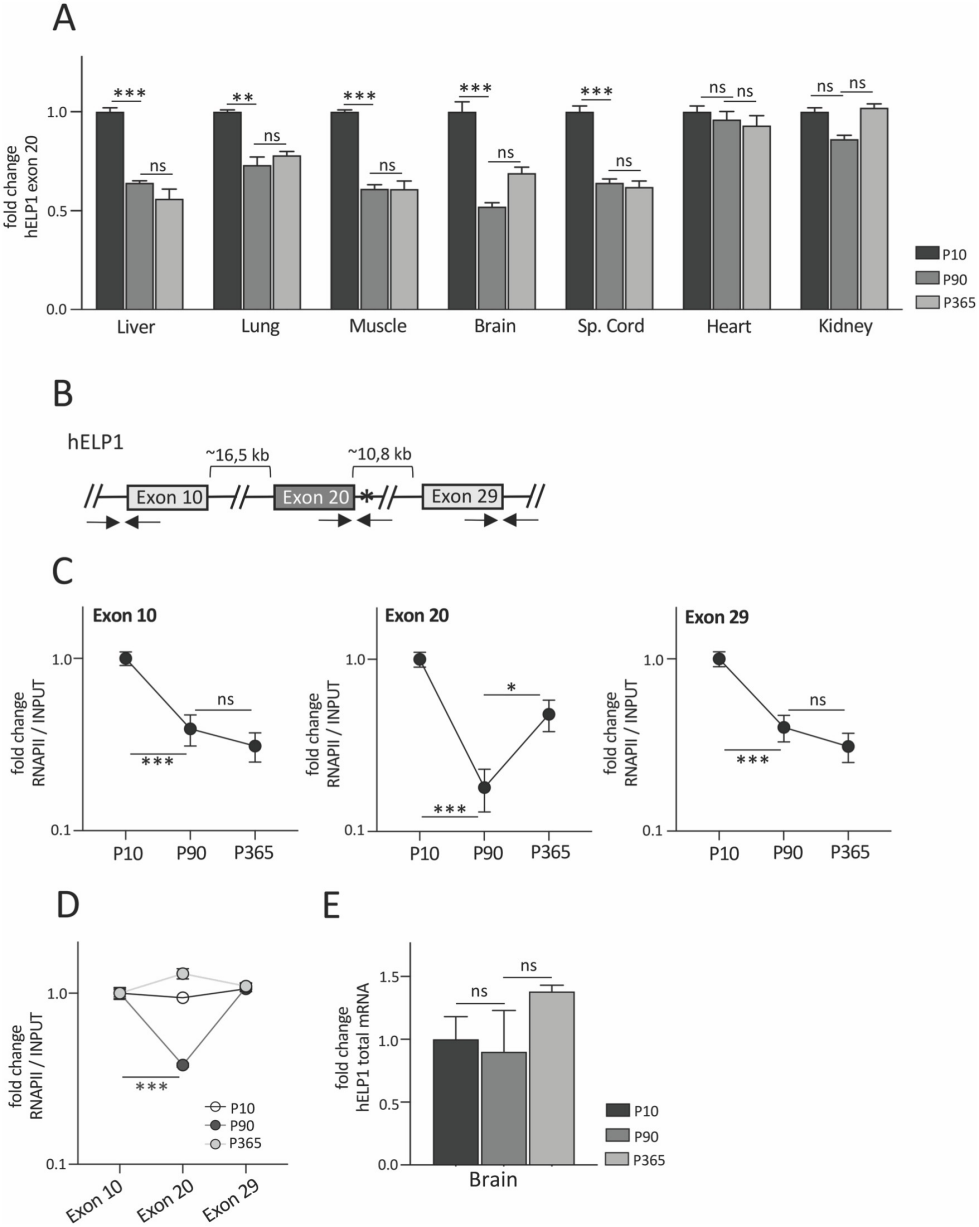
Temporal changes of hELP1 exon 20 splicing and RNA Polymerase II density profile in FD mouse. (**A**) Endpoint PCR of human ELP1 splicing isoforms in FD mouse model sacrificed at P10, P90 and P365. In each tissue, histogram displays the percentage (%) of ELP1 exon 20 inclusion expressed as fold change and the % of exon 20 inclusion of P10 animals is set to 1. (**B**) Schematic representation of the hELP1 gene: boxes represent exons whereas lines represent the sequence of the gene. The distance between exon 10 and exon 20 and exon 20 and exon 29, respectively, is indicate above in kilobases (kb). The c.2204+6T>C mutation is indicated as an asterisk and the position of the primers used for ChIP-qPCR is indicated below by arrows. (**C**) RNA Polymerase II (RNAPII) distribution on hELP1 exon 10, exon 20 and exon 29, respectively, assessed by ChIP-qPCR in brain of FD mouse model sacrificed at P10, P90 and P365. For each exon, data are expressed as fold change and the value of P10 animals is set to 1. (**D**) RNA Polymerase II (RNAPII) distribution along hELP1 gene body assessed by ChIP-qPCR in brain of FD mouse model sacrificed at P10, P90 and P365. For each time point, data are expressed as fold change and the value of exon 10 is set to 1. (**E**) Quantification by qRT-PCR of the total hELP1 mRNA expression levels in brain of FD mouse model sacrificed at P10, P90 and P365. The expression level of P10 animals is set to 1. In panels (A) and (E) data are expressed as mean ± s.e.m. of 3 mice for each age. In panels (C) and (D) data are expressed as mean + s.e.m. of n = 3 pooled mice with two technical replicates for each age and data are represented on an antilogarithmic scale. Statistical analysis was performed using OneWay ANOVA (no symbol or ns: not significant; * p<0.05; ** p<0.01; ***p<0.001).

To understand if RNAPII kinetic and chromatin dynamic can contribute to ELP1 exon 20 splicing regulation *in vivo*, we evaluated the temporal changes in brain tissue of a transgenic mouse model that carries the entire human ELP1 mutant gene (hELP1 c.2204+6T>C). This mouse model recapitulates the tissue-specific FD-ELP1 splicing pattern observed in FD patients but, due to the presence of the normal mouse IKBKAP gene, is phenotypically normal [43]. We initially evaluated splicing changes of ELP1 exon 20 in different tissues at 10, 90 and 365 post-natal days (P10, P90 and P365). During post-natal development (between P10 and P90), brain, spinal cord, liver, lung and muscle showed a significant decrease in the percentage of exon 20 inclusion. Brain and spinal cord, two nervous tissues affected in FD, have the highest levels of exon 20 skipping and showed a significant ~2-fold decrease in splicing between 10 and 90 days (Fig 2A and S2A and S2B Fig). Liver, lung, and muscle showed a ~1.6-, ~1.4- and ~1.6-folds decrease in exon 20 splicing, respectively, in the same period (Fig 2A and S2A and S2B Fig). At P365, brain, spinal cord, liver, lung, and muscle did not show any further change in exon 20 splicing (Fig 2A and S2A and S2B Fig). No age-dependent differences were observed for heart and kidney (Fig 2A and S2A and S2B Fig). Quantitative RT-PCR (qRT-PCR) analysis of exon 20 splicing showed similar temporal changes as detected by endpoint RT-PCR (ePCR) (S2C Fig). Due to the pathological role of the nervous system in FD, we focused on the brain where we assessed temporal changes in the rate of RNAPII elongation by chromatin immunoprecipitation followed by qPCR (ChIP-qPCR). The analysis was performed on the alternatively spliced exon 20 and on the constitutively spliced exons 10 and 29 which are ~16.5 kb and ~10.8 kb upstream and downstream of exon 20, respectively (Fig 2B). ChIP-qPCR analyses between P10 and P90 revealed a strong age-dependent reduction of RNAPII density for all exons: a ~2.5-, ~6- and ~2.5-folds decrease for exon 10, 20 and 29 respectively (Fig 2C). Between P90 and P365 RNAPII density slightly decreased for exons 10 and 29 but increased for the alternative spliced exon 20 (Fig 2C). It is plausible that in brain, between P90 and P365, the small increase in RNAPII rate and density does not reach the threshold level and therefore it is not sufficient to affect exon 20 splicing. The most pronounced decrease in RNAPII density was evident on exon 20 at P90: indeed, analysis of RNAPII enrichment throughout the hELP1 gene showed a ~2.5-folds decrease in RNAPII profile on alternatively spliced exon 20 at this age compared to the other exons (Fig 2D). These results indicate that during post-natal development (between P10 and P90) there is a general decrease in RNAPII density along the hELP1 gene, and that this decrease is more prominent on the alternative spliced exon 20. Since the total amount of hELP1 mRNA showed no alteration over time (Fig 2E), the age-dependent reduction in RNAPII density suggests an effect on RNAPII kinetics with a decrease in the rate of its elongation.

### Age-dependent changes of repressive H3K9me3 and H3K27me3 chromatin modifications in hELP1 gene in brain of FD transgenic mouse

**Fig 3:**
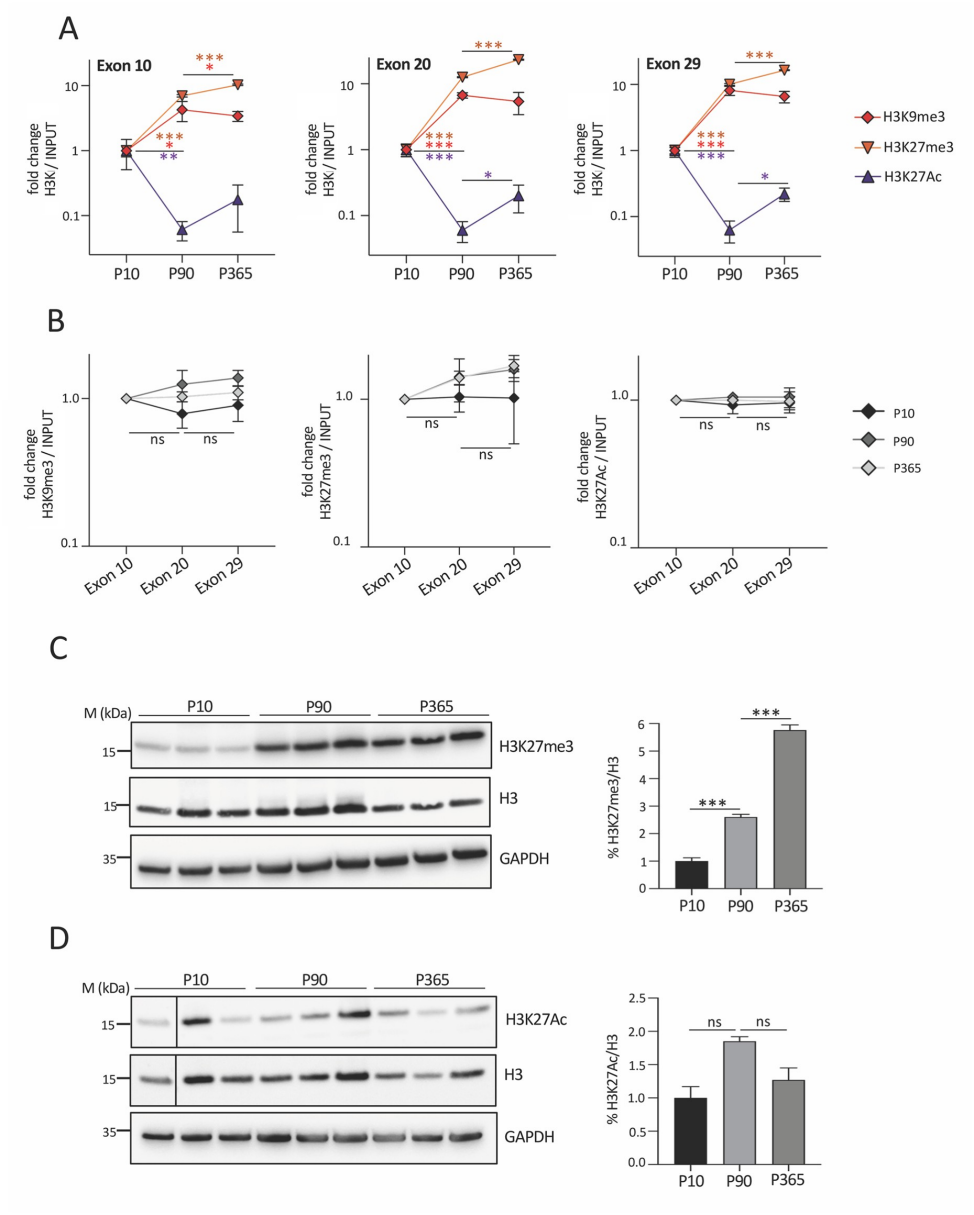
Temporal changes of H3K9me3, H3K27me3 and H3K27Ac density profile along hELP1 gene in FD mouse brain. (**A**) H3K9me3, H3K27me3 and H3K27Ac distribution on hELP1 exon 10, exon 20 and exon 29, respectively, assessed by ChIP-qPCR in brain of FD mouse model sacrificed at P10, P90 and P365. For each exon, data are expressed as fold change and the value of P10 animals is set to 1. (**B**) H3K9me3, H3K27me3 and H3K27Ac distribution along hELP1 gene body assessed by ChIP-qPCR in brain of FD mouse model sacrificed at P10, P90 and P365. For each time point, data are expressed as fold change and the value of exon 10 is set to 1. In panels (A) and (B) data are represented on an antilogarithmic scale. Immunoblotting analysis of total H3, (**C**) H3K27me3 and (**D**) H3K27Ac in brain of FD mice sacrificed at P10, P90 and P365. For H3K27me3 and H3K27Ac monoclonal antibodies were used; for total H3 polyclonal antibody was used. GAPDH was used as housekeeping loading control. The graphs represent the quantification analysis obtained with ImageJ software of the immunoblotting for (C) H3K27me3 and (D) H3K27Ac, respectively. The abundance of H3K27me3/H3 and H3K27Ac/H3 ratios of P10 pups is set to 1. Lines in panel (D) were drawn to align the first band with others. In panels (A) and (B) data are expressed as mean + s.e.m. of n = 3 pooled mice with two technical replicates for each age. In panels (C) and (D) data are expressed as mean ± S.D. of 3 mice for each age. Statistical analysis was performed using OneWay ANOVA (no symbol or ns: not significant; * p<0.05; ** p<0.01; ***p<0.001).

Since the RNAPII density profile can be modulated by changes in repressive chromatin marks [8], we focussed on H3K9me3 and H3K27me3, two histone lysines tri-methylation associated to a packaged chromatin conformation [60]. We performed ChIP-qPCR analyses in the brain of the FD model on ELP1 exons 10, 20 and 29 at P10, P90 and P365. Both H3K9me3 and H3K27me3 showed for the three exons a significant increase between P10 and P90 (Fig 3A). The most prominent variation between P10 and P90 was observed for the H3K27me3 repressive mark with a ~7- to ~13-folds increase. At P365, H3K27me3 further increased (~1.4- to ~1.8-folds) whereas H3K9me3 was not affected (Fig 3A). Since tri-methylation and acetylation on H3 lysine 27 residue are regulated in mutually exclusive manner [61, 62], we evaluated by ChIP-qPCR the age-dependent changes of H3K27Ac. Between P10 and P90, we found a sharp ~16-folds decrease of H3K27Ac (Fig 3A), diametrically opposite to the increase of the H3K27me3 profile. At later ages, between P90 and P365, H3K27Ac showed a small increase but still remained lower compared to P10 (Fig 3A). Next, we investigated if there is a preferential deposition of the histone marks over specific exons. With this analysis, we did not detect significant changes in the distribution pattern of the histone marks between the different exons at the different ages (Fig 3B). These results indicate that the temporal variations of H3K9me3, H3K27me3 and H3K27Ac in the brain are not specifically located at the alternative spliced exon 20 but occur along the entire hELP1 gene. To assess whether the age-dependent variations of H3K27me3 and H3K27Ac are specific of the hELP1 gene, we performed immunoblotting against these modifications in brain protein lysates at P10, P90 and P365. We observed a robust ~2.5-folds increase in the H3K27me3/H3 ratio during post-natal development and a similar ~2.4-folds enrichment, between P90 and P365 (Fig 3C), indicating that the age-dependent increase of the H3K27me3 mark on the hELP1 gene occurs in the entire tissue. Conversely, the global distribution of H3K27Ac in this tissue did not mimic its profile on exons 10, 20 and 29: though with some individual variability, we did not observe significant variations in the H3K27Ac/H3 ratio neither during post-natal development nor at later time-point (Fig 3D). As H3K27Ac is reduced on the ELP1 gene but does not change globally in the tissue, it is possible that this chromatin mark is specifically depleted on this gene in an age-dependent manner. All together these results demonstrate a profound rearrangement of the chromatin conformation along the entire hELP1 gene during postnatal development in the brain with a significant increase and decrease of H3K27me3 and H3K27Ac respectively, in the hELP1 gene body.

### Selective inhibition of H3K27me3 induces FD-ELP1 exon 20 inclusion and increases RNA Polymerase II elongation rate

**Fig 4:**
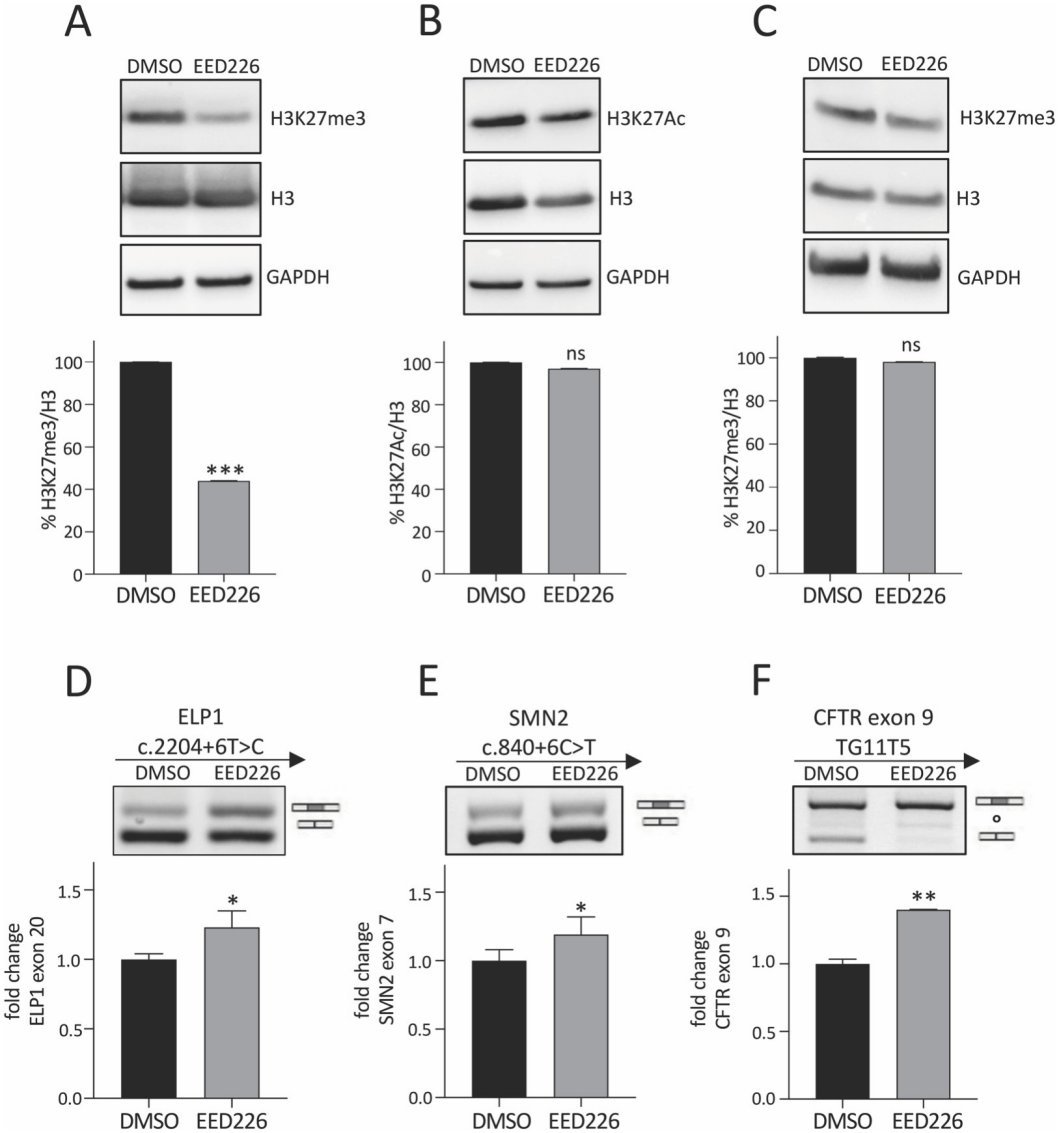
EED226 causes H3K27me3 reduction and enhances ELP1, SMN2 and CFTR aberrant splicing. Immunoblotting assay of total H3, (**A**) H3K27me3 and (**B**) H3K27Ac in HEK 293T cells and (**C**) H3K27me3 in FD-patients’ fibroblasts all treated with 0.1% DMSO or with EED226 100 uM for 24 h. For H3K27me3 and H3K27Ac, monoclonal antibodies were used; for total H3, polyclonal antibody was used. GAPDH were used as reference for internal normalization and representative samples are shown. The graph below represents the quantification analysis obtained with ImageJ software of the immunoblotting and the abundance of (A) H3K27me3/H3 and (B) H3K27Ac/H3 ratios of DMSO-treated cells is set to 100%. HEK 293T cells were transfected with (**D**) ELP1 mutant minigene, (**E**) SMN2 c.840+6C>T minigene and (**F**) CFTR exon 9 TG11T5 minigene and treated with 0.1% DMSO or EED226 100 uM for 24 h. DMSO-treated cells are set to 1. In panel (D) the ELP1 exon 20 inclusion and exclusion bands are indicated. In panel (E) the SMN2 exon 7 inclusion and exclusion bands are indicated. In panel (F) the CFTR exon 9 inclusion and exclusion bands are indicated and “°” denotes an already described cryptic splice site. In panels D—F, the intensity of the bands was measured with ImageJ software and histogram below gel displays the percentage of ELP1 exon 20, SMN2 exon 7 and CFTR exon 9 inclusion, respectively, expressed as fold change. DMSO-treated cells are set to 1. Data are expressed as mean + S.D. of n = 3 experiments in triplicate. Statistical analysis was performed using Student t-test (ns: not significant; *p<0.05; **p<0.01; *** p<0.001).

**Fig 5:**
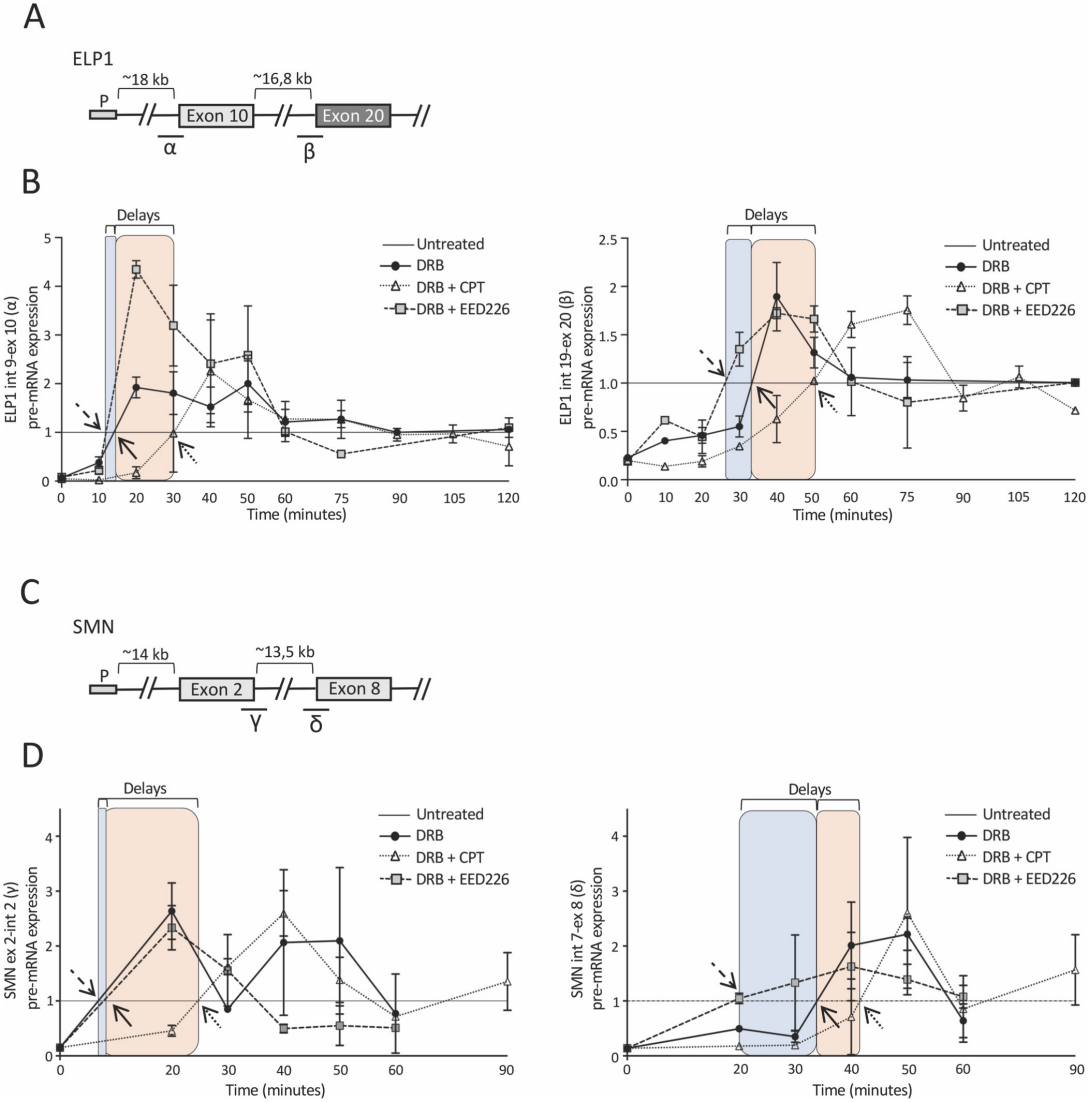
EED226 and CPT modulate RNA Polymerase II speed along ELP1 and SMN genes. (**A**) Schematic representation of the ELP1 gene: boxes represent promoter (P), exons 10 and 20 whereas lines represent the sequence of the gene. The distances between the promoter and exon 10 and exons are indicated above in kilobases (kb). Regions for RNAPII-elongation analyses are indicated below (α–β). (**B**) Analysis of transcriptional elongation of the regions indicated in panel (A) in the presence or absence of CPT 1uM or EED226 100 uM. (**C**) Schematic representation of the SMN genes: boxes represent promoter (P), exons 2 and 8 whereas lines represent the sequence of the gene. The distances between the promoter and exon 2 and exons are indicated above in kilobases (kb). Regions for RNAPII-elongation analyses are indicated below (γ–δ). (**D**) Analysis of transcriptional elongation of the regions indicated in panel (C) in the presence or absence of CPT 1uM or EED226 100 uM. For both genes, the values are relativized to the pre-mRNA levels of DRB-treated cells and normalized to U6 snRNA expression. Arrows indicate the intersect between DRB (black arrow), DRB+EED226 (traced arrow) and DRB+CPT (dotted arrow) kinetic profiles, respectively, with control (untreated) and colored rectangles represent the kinetic profiles’ delay between DRB, DRB+EED226 (in blue) and DRB+CPT (in pink), respectively. Data are expressed as mean + s.e.m. of n = 2 independent measurements.

To evaluate the potential role of H3K27me3 on splicing regulation, we tested EED226. This antitumor small molecule is an allosteric and selective Polycomb Repressive Complex 2 (PRC2) inhibitor that binds to the H3K27me3 pocket of EED, leading to loss of PRC2 activity and causing a specific H3K27 methylation reduction [63, 64]. We evaluated by immunoblotting with specific antibodies the effect of EED226 on H3K27me3 and H3K27Ac protein levels in HEK 293T cells and FD fibroblasts. In HEK 293T cells, we observed a significant reduction of approximately 60% in the H3K27me3/H3 ratio (Fig 4A) and no change in the H3K27Ac/H3 ratio (Fig 4B). In FD fibroblasts, EED226 did not change the H3K27me3/H3 ratio (Fig 4C) and the ELP1 exon 20 splicing pattern (S3A Fig) suggesting that this antitumor drug acts preferentially on cancer-derived cells while sparing primary cells. To evaluate the effect of the drug on splicing we initially performed minigene experiments with FD-ELP1 exon 20, SMN2 exon 7 and CFTR exon 9. The latter two disease-associated exons have been shown to be regulated by RNAPII kinetics and/or chromatin status [14, 15]. Transfection experiments showed that the EED226-mediated inhibition of H3K27me3 induce a small but significant increase in the exon inclusion levels. ELP1 and SMN2 showed a ~1.2- folds increase in exon 20 and exon 7 inclusion, respectively, whereas CFTR exon 9 levels increased ~1.4- folds (Fig 4D–4F) [65]. EED226 had no or a moderate effect on other alternative spliced exons (FN1 exon 25, FN1 exon 33 and FIX exon 5Δ6) (S3B–S3D Fig) [11, 66] and did not affect ELP1 exon 10 and exon 20 and CFTR exon 9 constitutive exons (S3F–S3H Fig), suggesting a H3K27me3 selective effect on alternative splicing. To elucidate if the effect of H3K27me3 on splicing can be due to changes in RNAPII kinetics, we performed an elongation assay [14, 23, 28]. We evaluated the endogenous pre-mRNA enrichment in EED226-treated HEK 293T cells at different time points after DRB wash focusing on the transcripts originated from the ELP1 and SMN endogenous genes (Fig 5A and 5C). CPT-treated cells were used as control, since a previous study has shown that CPT slows down RNAPII rate on CFTR gene [14]. Upon EED226 addition, we found an earlier transcription recovery time for all amplicons (Fig 5B and 5D). Compared to untreated cells, EED226 induced the nascent ELP1 exon 10 transcripts to appear ~3 minutes earlier, which increased to ~10 minutes for the most distal exon 20 (Fig 5B, blue rectangles). A similar increase in RNAPII speed was observed on the SMN gene (Fig 5D, blue rectangles). Control CPT treatment, reducing the speed of RNAPII, induces a delay of the amplification recovery (Fig 5B and 5D, pink rectangles). These results indicate that the EED226-mediated inhibition of H3K27me3 removes a roadblock to the elongating RNAPII increasing its speed, which in turn affect splicing of responsive alternative exons.

### H3K27me3 modulates alternative splicing in a global manner

**Fig 6:**
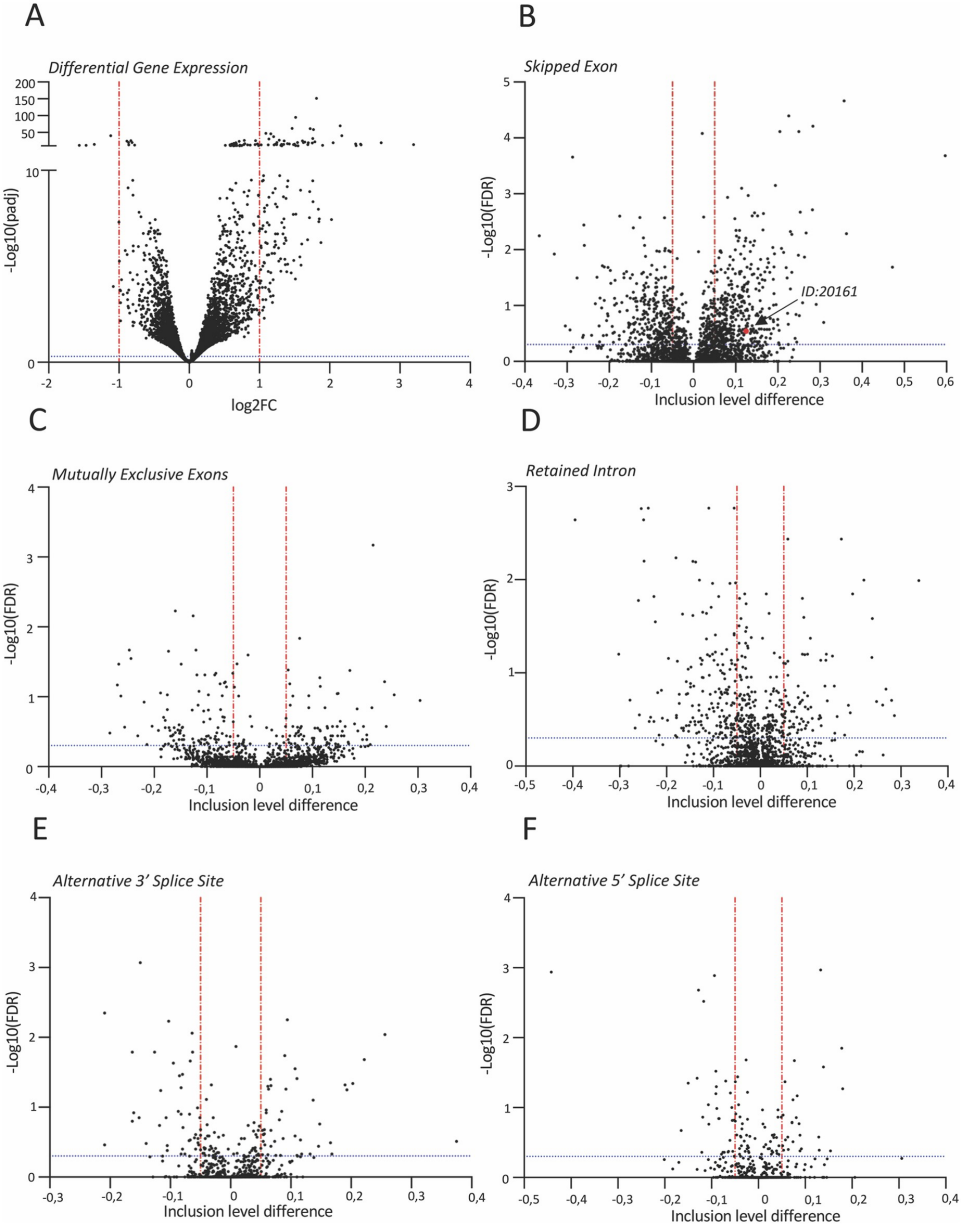
Effects of EED226 on human transcriptome. (**A**) Volcano plot showing global differential gene expression changes between HEK 293T cells (n = 3) and cells treated with EED226 100 uM for 48 h (n = 3). Horizontal blue line (padj ≤ 0.5) and vertical red lines (log_2_FoldChange ≤ -1 or ≥ 1) indicate cut-off values and determine significant up- and down-regulated events, respectively. Volcano plots showing global alternative splicing changes in (**B**) skipped exon (SE), (**C**) mutually exclusive exons (MXE), (**D**) retained intron (RI), (**E**) alternative 3’ splice site (A3SS) and (**F**) alternative 5’ splice site (A5SS) categories between HEK 293T cells (n = 3) and cells treated with EED226 100 uM for 48 h (n = 3). In each plot, horizontal blue line (FDR ≤ 0.5) and vertical red lines (inclusion level difference ≤ -0.05 or ≥ 0.05) indicate cut-off values and determine significant events. The red dot in panel (B) corresponds to SMN2 exon 7 (ID = 20161).

To evaluate the role of H3K27me3 on alternative splicing globally, we performed RNA sequencing analysis in the EED226-treated cells. We found 143 differentially expressed genes (DEG) out of 14165 transcripts (1,01%) (mean counts ≥ 50, log_2_ fold change ≤ -1 or ≥ 1), mostly upregulated (138 up and 5 down) (Fig 6A) (S3 Table). This preferential upregulation is consistent with the established repressive role of H3K27me3 at promoters [67–69]. Analysis of the differentially alternative splicing identified 728 events out of 22075 transcripts (3,30%) for skipped exons (SE) (408 up- and 320 down-regulated, respectively) (Fig 6B) (S4 Table). This group also includes endogenous SMN2 exon 7 (ID: 20161) whose inclusion was confirmed by qRT-PCR analysis (S4 Fig). ELP1 exon 20 and CFTR exon 9 were not detected as HEK 293T cells have normal ELP1 gene and do not express *CFTR*. Mutually exclusive exons (MXE) showed 181 events out of 2637 transcripts (6,86%), retained introns (RI) 253 events out of 2354 transcripts (10,75%) and alternative 3’ and 5’ splice sites showed 90 out of 1656 (5,4%) and 55 out of 1155 transcripts (4,7%), respectively (Fig 6C–6F) (S2 Table) (S5–S8 Tables). H3K27me3 depletion did not affect those alternatively spliced genes involved in cell migration and invasion (*FGFR2*, *CTNND1*, *TCF7L2*, *SLK*, *SCRIB*) whose alternative splicing depends on subsequent PTB recruitment on H3K27 marks [28] (S9 Table). As drugs that inhibit RNAPII elongation might activate pathways involved in splicing control [58], we tested DEG using Qiagen’s Ingenuity Pathway Analysis (IPA). We did not detect changes related to splicing/pre-mRNA processing (S10 Table), strongly suggesting that the major effect of H3K27me3 depletion on splicing is due to changes in RNAPII transcriptional elongation. In addition, there are no genes in common between DEGs and differentially alternative splicing indicating that release of transcript repression of the H3K27me3 mark at promoters does not directly affect alternative splicing events of the same genes.

## Discussion

Despite the established contribution of splicing defects to human disease, very few pathological cases have been described as being regulated by RNAPII kinetics and/or by chromatin architecture. In this paper, we show that FD-ELP1 exon 20, which is the splicing event defective in Familial Dysautonomia, follows the kinetic model of co-transcriptional splicing and, like SMN2 exon 7, belongs to the type II alternative exons, being positively affected by the RNAPII elongation rate. We also provide compelling evidence that H3K27me3/Ac acts as an important splicing-regulatory chromatin mark that, deposited on the FD-ELP1 gene body, modulates RNAPII speed and affects the ELP1 exon 20 inclusion. With a direct elongation assay, we show that H3K27me3 depletion increases RNAPII rate on ELP1 and SMN genes and promotes inclusion of corresponding alternative exons, evidence that indicates a direct link between the chromatin mark, RNAPII speed and alternative splicing. In the brain of an FD mouse model, a substantial age-dependent increase in the H3K27me3/Ac chromatin ratio and changes in RNAPII elongation rate correlate with a decrease in ELP1 exon 20 splicing, particularly in the first months of life. This result suggests that changes in H3K27me3/Ac modify the rate of RNAPII elongation which in turn regulates alternative splicing of FD ELP1 exon 20. H3K27me3/Ac are important regulatory marks at the promoter of responsive genes where they have an established role in modulating transcription [61, 62, 67–69]. Besides, H3K27me3 belongs to a group of splicing-associated chromatin signatures that, located in the body of genes, defines the level of inclusion of a subset of alternative spliced exons [70]. H3K27me3/Ac along with other histone modifications regulates splicing of NCAM exon 18 during neuronal cells depolarization and differentiation [19, 23] and FGFR2 mutually exclusive exons IIIb/IIIc during epithelial/mesenchymal cells transition [21]. These signatures have also been involved in the regulation of AS during human embryonic stem cells differentiation [71] and tumour progression [28, 72]. We show here that H3K27me3 negatively regulates three disease associated splicing defects in ELP1, SMN2 and CFTR genes but not in other cases (FN1 exon 25, FN1 exon 33 and FIX exon 5Δ6) and, by means of RNA-Seq, that this mark has a global effect on a relatively large group of alternative splicing events. These H3K27me3 sensitive exons might be specifically enriched with this histone signature or have a unique local chromatin architecture (e.g., free from antagonist chromatin marks). In accordance with the kinetic coupling model [9–15], depletion of H3K27me3, by increasing RNAPII elongation, can affect exon (SE) in both directions depending on the type of regulatory sequences that gradually emerge from pre-mRNA transcript. Indeed, we observed that after EED226 treatment a similar number of SE events were up- and down- regulated (408 and 320, respectively) (Fig 6B). While we have not addressed why ELP1 exon 20 is negatively regulated by RNAPII rate, the presence of negative regulatory elements that bind to HNRNPA1 [73], similar to SMN2, might be involved [15]. The substantial number of retained introns (RI) (253 events) we detected by RNA-Seq is also consistent with a reduced time for intron processing by an increased RNAPII elongation (Fig 6D). To explore the role of co-transcriptional splicing regulation *in vivo* we focused on the FD-ELP1 mouse brain where we observed a temporal decrease in ELP1 exon 20 inclusion levels (Fig 2A and S2A Fig). If this also occurred in FD patients, it could have negative functional consequences contributing to aggravate the disease phenotype at an early age, providing an additional reason to start corrective therapies as soon as possible. While we cannot rule out other mechanisms, our results indicate that chromatin may contribute to the temporal decrease of ELP1 exon 20 inclusion. In line with this, we observed that reduced ELP1 exon 20 splicing is associated with a decrease in the RNAPII density profile on the whole human FD-ELP1 transgene (Fig 2C), a large increase in the two chromatin repressive marks H3K27me3 and H3K9me3 and a decrease of H3K27Ac (Fig 3A). H3K9me3 and H3K27me3 are structural silencing marks that are dynamically modified during development and differentiation [74]. H3K9me3 is a permanent repression signal designed for the heterochromatin formation [75] and has been associated to aging in different species [76–79]. H3K27me3 is closely associated with a set of regulators genes expressed during development [80]. An age-dependent increase in H3K27me3 has been observed in human peripheral blood mononuclear cells [81], in human adipose-derived mesenchymal stem cells [82, 83] and in quiescent mouse muscle stem cells [79], in brain and skeletal muscle of the killfish torquoise *Nothobranchius furzeri* [84, 85] and in the brain of SAMP8 mice, a strain of mice that displays a phenotype of accelerated aging [86]. In *Drosophila melanogaster*, decrease in the H3K27me3 status increases the flies’ longevity [87] whereas in *Caenorhabditis elegans*, a decrease in H3K27me3 levels is associated with premature aging [88]. Since we show here that H3K27me3 influences pre-mRNA processing through RNAPII kinetics, it would be interesting to explore the contribution of alternative splicing in these different cases as well. Interestingly, the age-dependent increase of the H3K27me3 mark on the hELP1 gene (Fig 3A) is reflected by an increase in the entire brain tissue (Fig 3C) suggesting that this chromatin modification might affect multiple splicing events. Because of the interesting similarity between the co-transcriptional regulation of ELP1 exon 20 in FD and SMN2 exon 7 in SMA [15], both of which are positively regulated by RNAPII elongation rate, our results have interesting implication for the development of new strategies for splicing correction based on chromatin modification. In a SMA mouse model, the chromatin-modifying compound VPA, which is approved by FDA for human treatment, has been shown, by improving SMN2 exon 7 inclusion, to counteract the unexpected negative effect that ASOs have on chromatin [15]. Because splicing correction strategies based on ASO have been also identified in FD [45], chromatin-modifying compound as VPA might also be considered for this disease. Interestingly, our results indicate that inclusion of ELP1 exon 20 and SMN2 exon 7 is activated by drugs that specifically target H3K27me3 (Fig 4D and 4E), with enhancement of RNAPII elongation (Fig 5). EED226 is a first-in-class EED inhibitor that has been shown to possess a strong antitumor activity in xenograft mouse models [89] and it is under evaluation in a phase 1/2 clinical trial for advanced malignancies (NCT02900651) [90]. This chemical exerts its anti-tumour activity by selectively binding to the H3K27me3 binding pocket of EED protein, a subunit of PRC2, and allosterically inhibits PRC2 complex [63]. EED226-mediated inhibition of H3K27me3 influences some AS exons (ELP1, SMN2, CFTR and FIX but not FN1 exon 25 and exon 33) (Fig 4D–4F and S3B–S3D Fig). However, as this antitumour drug seems to reduce H3K27me3 preferentially on cancer-derived cells while sparing primary cells its potential application as splicing modulator needs to be clarified. In general changes in RNAPII elongation speed induced by mutant RNAPIIs [17, 58], by different chemicals (CPT, DRB, VPA or TSA) [14, 15, 58] or chromatin alterations [15, 19, 23], do not have major effect on constitutive splicing. Also, in our RNA-seq experiments with EED226-mediated inhibition of H3K27me3 we did not observe an effect on constitutive splicing. Thus, changes in RNAPII elongation or chromatin that occur along the entire body of a gene preferentially targets AS exons, sparing the constitutive ones. In brain, between P10-P90 and P10-P365, we observed for H3K27me3 a strong 7- to 23-folds increase along the hELP1 gene (Fig 3A) and a 6-folds increase in the tissue (Fig 3C). This chromatin rearrangement is associated with a general 2.5- to 6-folds decrease in the RNAPII density (Fig 2C) and no specific enrichment of H3K27me3 over the alternative spliced exon (Fig 3B). In cellular model, a more direct evaluation of the RNAPII speed shows that H3K27me3 depletion by EED226 accelerates the RNAPII elongation on two target genes, SMN and ELP1 (Fig 5), modifying splicing of the AS exons and not the constitutive ones. Thus, we propose that models that consider the effect of RNAPII kinetics on splicing regulation should consider not only changes occurring at AS regions but also differences along the entire gene body. Compared to the substantial numbers of permissive and repressive histone modifications identified, the chromatin changes that have been reported to regulate splicing [15, 19, 23, 70] probably represent just the tip of the iceberg. Here we have mainly focused on H3K27me3, as it shows an impressive 13-folds temporal increase in brain development, but it is reasonable to think that changes in many other chromatin marks with potential effect on splicing are common during development, aging or cancer. As these chromatin changes, either affecting the RNAPII elongation (as we show here or in [15, 20, 23]) and/or modulating splicing factors recruitment [28, 91], can have an antagonistic splicing activity, the final splicing decision will be the result of the sum of their multiple effects. Considering that the most studied classical pathways based on splicing factors, which are not sufficient to fully clarify the final splicing decision, the elucidation of the complex interplay between chromatin marks and splicing might help in providing a more precise understanding of alternative splicing.

## Supporting information

S1 Fig RNA Polymerase II elongation rate and chromatin structure do not affect splicing of constitutive spliced exons. (**A**) HEK 293T cells were transfected with ELP1 mutant minigene (c.2204+6T>C) and treated with 0.1% DMSO or CPT 12 uM or DRB 75 uM for 24 h. DMSO-treated cells are set to 1. (**B**) HEK 293T cells were treated with 0.1% DMSO or CPT 12 uM or DRB 75 uM for 24 h. DMSO-treated cells are set to 1. (**C**) HEK 293T cells were treated with 0.1% DMSO or CPT 12 uM or DRB 75 uM for 24 h. DMSO-treated cells are set to 1. **(D**) Healthy donor (WT) fibroblasts were treated with 0.1% DMSO or CPT 12 uM or DRB 20 uM for 24 h. DMSO-treated cells are set to 1. (**E**) HEK 293T cells were co-transfected with ELP1 mutant minigene and the expression vectors for WTresPol II and R749H slow mutant RNA Polymerase II followed by the addition of 2.5 ug/mL or 5 ug/mL of α-amanitin for 24h. Cells transfected only with ELP1 mutant minigene and treated with 0.1% DMSO are set to 1. (**F**) HEK 293T cells were co-transfected with the expression vectors for WTresPol II and R749H slow mutant RNAPII followed by the addition of 2.5 ug/mL or 5 ug/mL of α-amanitin for 24h. DMSO-treated are set to 1. (**G**) HEK 293T cells were transfected with ELP1 mutant minigene (c.2204+6T>C) and treated with 0.1% DMSO or VPA 4 mM or TSA 1 ug/mL for 24 h. DMSO-treated cells are set to 1. (**H**) HEK 293T cells were treated with 0.1% DMSO or VPA 4 mM or TSA 1 ug/mL for 24 h. DMSO-treated cells are set to 1. (**I**) HEK 293T cells were treated with 0.1% DMSO or VPA 4 mM or TSA 1 ug/mL for 24 h. DMSO-treated cells are set to 1. (**J**) Healthy donor (WT) fibroblasts were treated with 0.1% DMSO or VPA 8 mM or TSA 1 ug/mL for 24 h. DMSO-treated cells are set to 1. In all panels, HBA exon 2, ELP1 exon 10 and ELP1 exon 20 inclusion bands are indicated and in panels (A), (E) and (G) the HBA upper band corresponds to intron 1 retention. The intensity of the bands was measured with ImageJ software and histogram below gel displays the percentage of exon inclusion expressed as fold change. Dividing lines indicate cropping and annealing of the same agarose gel or experiment. Data are expressed as mean + S.D. of n = 3 experiments in triplicate. Statistical analysis was performed using Student t-test (ns: not significant).
(TIF)

S2 Fig hELP1 exon 20 reveals a temporal splicing pattern in FD mouse tissues. (**A**) Endpoint PCR of hELP1 splicing pattern in FD mouse sacrificed at P10, P90 and P365. Identity of exon inclusion (202 bp) and skipping (128 bp) bands are indicated on the right and tissues analyzed on the left of the gel. (**B**) Quantification of the intensity of ePCR gels bands with ImageJ software. Data are expressed as percentage of hELP1 exon 20 inclusion. (**C**) SYBR green based-qPCR quantification of the ratio of hELP1 FL (mRNAs including exon 20) to Δ20 (mRNAs lacking exon 20) transcripts in FD mouse model sacrificed at P10, P90 and P365. In each tissue, the expression level of P10 animals is set to 1. Data are expressed as mean + s.e.m. of 3 mice for each age. Statistical analysis was performed using OneWay ANOVA (ns: not significant; * p<0.05; ** p<0.01; ***p<0.001).
(TIF)

S3 Fig EED226 treatment effect on splicing of WT ELP1 exon 20, FN1, FIX and constitutive spliced exons. (**A**) FD patients’ fibroblasts were treated with 0.1% DMSO or EED226 100 uM for 24 h. DMSO-treated cells are set to 1. HEK 293T cells were transfected with (**B**) FN1 exon 33 (EDA) minigene, (**C**) FN1 exon 25 (EDB) minigene and (**D**) FIX exon 5Δ6 minigene and treated with 0.1% DMSO or EED226 100 uM for 24 h. DMSO-treated cells are set to 1. (**E**) HEK 293T cells were transfected with ELP1 mutant minigene (c.2204+6T>C) and treated with 0.1% DMSO or EED226 100 uM for 24 h. DMSO-treated cells are set to 1. (**F**) HEK 293T cells were treated with 0.1% DMSO or EED226 100 uM for 24 h. DMSO-treated cells are set to 1. (**G**) HEK 293T cells were treated with 0.1% DMSO or EED226 100 uM for 24 h. DMSO-treated cells are set to 1. (**H**) HEK 293T cells were transfected with CFTR exon 9 wt minigene and treated with 0.1% DMSO or EED226 100 uM for 24 h. DMSO-treated cells are set to 1. # denotes PCR artifacts. In panel (B), FN1 EDA inclusion and exclusion bands are indicated. In panel (C), FN1 EDB inclusion and exclusion bands are indicated. In panel (D), FIX exon 5Δ6 inclusion and exclusion bands are indicated. In panel (E), HBA exon 2 inclusion band is indicated and the HBA upper band corresponds to intron 1 retention. In panels (F), (G) and (H) ELP1 exon 10, ELP1 exon 20 wt and CFTR exon 9 wt inclusion bands are indicated, respectively. In all panels, the intensity of the bands was measured with ImageJ software and histogram below gel displays the percentage of exon inclusion expressed as fold change. Data are expressed as mean + S.D. of n = 3 experiments in triplicate. Statistical analysis was performed using Student t-test (ns: not significant; * p< 0.05).
(TIF)

S4 Fig EED226 treatment increases SMN2 exon 7 inclusion in HEK 293T cells. HEK 293T cells were treated with 0.1% DMSO or EED226 100uM for 48 h and RT-PCR amplified fragments digested with DdeI restriction enzyme to obtain SMN1 and SMN2 exon 7 inclusion (FL) and exclusion (Δ7) fragments, respectively. The SMN2 exon 7 inclusion and exclusion bands and a fragment of exon 8 are indicated. DMSO-treated cells are set to 1. The intensity of the bands was measured with ImageJ software and histogram below gel displays the percentage of exon inclusion expressed as fold change. Data are expressed as mean + S.D. of n = 3 experiments in triplicate. Statistical analysis was performed using Student t-test (* p<0.05).
(TIF)

S1 Table Oligonucleotides used in this study. (PDF)

S2 Table Summary table of alternative splicing analysis in HEK 293T cells treated with EED226. (PDF)

S3 Table Differentially expressed genes in HEK 293T treated with EED226 vs control. (XLS)

S4 Table Splicing changes in skipped exon (SE) category in HEK 293T treated with EED226 vs control. (XLS)

S5 Table Splicing changes in mutually exclusive exons (MXE) category in HEK 293T treated with EED226 vs control. (XLS)

S6 Table Splicing changes in retained introns (RI) category in HEK 293T treated with EED226 vs control. (XLS)

S7 Table Splicing changes in alternative 3’ splice sites (A3SS) category in HEK 293T treated with EED226 vs control. (XLS)

S8 Table Splicing changes in alternative 5’ splice sites (A5SS) category in HEK 293T treated with EED226 vs control. (XLS)

S9 Table List of selected AS events identified in Segelle et al., 2022. (XLS)

S10 Table List of canonical pathways in HEK 293T treated with EED226 vs control. (XLS)

S1 File Raw images of gels shown in this study. (PDF)

## References

[1] Ast G. How did alternative splicing evolve? Nat Rev Genet. 2004;5: 773–782. doi: 10.1038/nrg1451 15510168

[2] Baralle FE, Giudice J. Alternative splicing as a regulator of development and tissue identity. Nat Rev Mol Cell Biol. 2017;18: 437–451. doi: 10.1038/nrm.2017.27 28488700 PMC6839889

[3] Naftelberg S, Schor IE, Ast G, Kornblihtt AR. Regulation of Alternative Splicing Through Coupling with Transcription and Chromatin Structure. Annu Rev Biochem. 2015;84: 165–198. doi: 10.1146/annurev-biochem-060614-034242 26034889

[4] Matera AG, Wang Z. A day in the life of the spliceosome. Nat Rev Mol Cell Biol. 2014;15: 108–121. doi: 10.1038/nrm3742 24452469 PMC4060434

[5] Ule J, Blencowe BJ. Alternative Splicing Regulatory Networks: Functions, Mechanisms, and Evolution. Mol Cell. 2019;76: 329–345. doi: 10.1016/j.molcel.2019.09.017 31626751

[6] Fu X-D, Ares M. Context-dependent control of alternative splicing by RNA-binding proteins. Nat Rev Genet. 2014;15: 689–701. doi: 10.1038/nrg3778 25112293 PMC4440546

[7] Gómez Acuña LI, Fiszbein A, Alló M, Schor IE, Kornblihtt AR. Connections between chromatin signatures and splicing: Connections between chromatin signatures and splicing. WIREs RNA. 2013;4: 77–91. doi: 10.1002/wrna.1142 23074139

[8] Giono LE, Kornblihtt AR. Linking transcription, RNA polymerase II elongation and alternative splicing. Biochem J. 2020;477: 3091–3104. doi: 10.1042/BCJ20200475 32857854

[9] Kadener S, Cramer P, Nogués G, Cazalla D, de la Mata M, Fededa JP, et al. Antagonistic effects of T-Ag and VP16 reveal a role for RNA pol II elongation on alternative splicing. EMBO J. 2001;20: 5759–5768. doi: 10.1093/emboj/20.20.5759 11598018 PMC125675

[10] Nogues G, Kadener S, Cramer P, Bentley D, Kornblihtt AR. Transcriptional activators differ in their abilities to control alternative splicing. J Biol Chem. 2002;277: 43110–43114. doi: 10.1074/jbc.M208418200 12221105

[11] de la Mata M, Alonso CR, Kadener S, Fededa JP, Blaustein M, Pelisch F, et al. A Slow RNA Polymerase II Affects Alternative Splicing In Vivo. Molecular Cell. 2003;12: 525–532. doi: 10.1016/j.molcel.2003.08.001 14536091

[12] Kornblihtt AR. Multiple links between transcription and splicing. RNA. 2004;10: 1489–1498. doi: 10.1261/rna.7100104 15383674 PMC1370635

[13] Kornblihtt AR. Coupling transcription and alternative splicing. Adv Exp Med Biol. 2007;623: 175–189. doi: 10.1007/978-0-387-77374-2_11 18380347

[14] Dujardin G, Lafaille C, de la Mata M, Marasco LE, Muñoz MJ, Le Jossic-Corcos C, et al. How Slow RNA Polymerase II Elongation Favors Alternative Exon Skipping. Molecular Cell. 2014;54: 683–690. doi: 10.1016/j.molcel.2014.03.044 24793692

[15] Marasco LE, Dujardin G, Sousa-Luís R, Liu YH, Stigliano JN, Nomakuchi T, et al. Counteracting chromatin effects of a splicing-correcting antisense oligonucleotide improves its therapeutic efficacy in spinal muscular atrophy. Cell. 2022;185: 2057–2070.e15. doi: 10.1016/j.cell.2022.04.031 35688133 PMC9555286

[16] Rogalska ME, Vivori C, Valcárcel J. Regulation of pre-mRNA splicing: roles in physiology and disease, and therapeutic prospects. Nat Rev Genet. 2022. doi: 10.1038/s41576-022-00556-8 36526860

[17] Fong N, Kim H, Zhou Y, Ji X, Qiu J, Saldi T, et al. Pre-mRNA splicing is facilitated by an optimal RNA polymerase II elongation rate. Genes Dev. 2014;28: 2663–2676. doi: 10.1101/gad.252106.114 25452276 PMC4248296

[18] Maslon MM, Braunschweig U, Aitken S, Mann AR, Kilanowski F, Hunter CJ, et al. A slow transcription rate causes embryonic lethality and perturbs kinetic coupling of neuronal genes. EMBO J. 2019;38: e101244. doi: 10.15252/embj.2018101244 30988016 PMC6484407

[19] Schor IE, Rascovan N, Pelisch F, Alló M, Kornblihtt AR. Neuronal cell depolarization induces intragenic chromatin modifications affecting NCAM alternative splicing. Proc Natl Acad Sci U S A. 2009;106: 4325–4330. doi: 10.1073/pnas.0810666106 19251664 PMC2657401

[20] Alló M, Buggiano V, Fededa JP, Petrillo E, Schor I, de la Mata M, et al. Control of alternative splicing through siRNA-mediated transcriptional gene silencing. Nat Struct Mol Biol. 2009;16: 717–724. doi: 10.1038/nsmb.1620 19543290

[21] Luco RF, Pan Q, Tominaga K, Blencowe BJ, Pereira-Smith OM, Misteli T. Regulation of Alternative Splicing by Histone Modifications. Science. 2010;327: 996–1000. doi: 10.1126/science.1184208 20133523 PMC2913848

[22] Saint-André V, Batsché E, Rachez C, Muchardt C. Histone H3 lysine 9 trimethylation and HP1γ favor inclusion of alternative exons. Nat Struct Mol Biol. 2011;18: 337–344. doi: 10.1038/nsmb.1995 21358630

[23] Schor IE, Fiszbein A, Petrillo E, Kornblihtt AR. Intragenic epigenetic changes modulate NCAM alternative splicing in neuronal differentiation. EMBO J. 2013;32: 2264–2274. doi: 10.1038/emboj.2013.167 23892457 PMC3746202

[24] Sims RJ, Millhouse S, Chen C-F, Lewis BA, Erdjument-Bromage H, Tempst P, et al. Recognition of trimethylated histone H3 lysine 4 facilitates the recruitment of transcription postinitiation factors and pre-mRNA splicing. Mol Cell. 2007;28: 665–676. doi: 10.1016/j.molcel.2007.11.010 18042460 PMC2276655

[25] Piacentini L, Fanti L, Negri R, Del Vescovo V, Fatica A, Altieri F, et al. Heterochromatin protein 1 (HP1a) positively regulates euchromatic gene expression through RNA transcript association and interaction with hnRNPs in Drosophila. PLoS Genet. 2009;5: e1000670. doi: 10.1371/journal.pgen.1000670 19798443 PMC2743825

[26] Gunderson FQ, Johnson TL. Acetylation by the transcriptional coactivator Gcn5 plays a novel role in co-transcriptional spliceosome assembly. PLoS Genet. 2009;5: e1000682. doi: 10.1371/journal.pgen.1000682 19834536 PMC2752994

[27] Loomis RJ, Naoe Y, Parker JB, Savic V, Bozovsky MR, Macfarlan T, et al. Chromatin binding of SRp20 and ASF/SF2 and dissociation from mitotic chromosomes is modulated by histone H3 serine 10 phosphorylation. Mol Cell. 2009;33: 450–461. doi: 10.1016/j.molcel.2009.02.003 19250906 PMC2667802

[28] Segelle A, Núñez-Álvarez Y, Oldfield AJ, Webb KM, Voigt P, Luco RF. Histone marks regulate the epithelial-to-mesenchymal transition via alternative splicing. Cell Rep. 2022;38: 110357. doi: 10.1016/j.celrep.2022.110357 35172149

[29] Yano M, Hayakawa-Yano Y, Mele A, Darnell RB. Nova2 regulates neuronal migration through an RNA switch in disabled-1 signaling. Neuron. 2010;66: 848–858. doi: 10.1016/j.neuron.2010.05.007 20620871 PMC2965850

[30] Linke WA, Krüger M. The giant protein titin as an integrator of myocyte signaling pathways. Physiology (Bethesda). 2010;25: 186–198. doi: 10.1152/physiol.00005.2010 20551232

[31] Guo W, Schafer S, Greaser ML, Radke MH, Liss M, Govindarajan T, et al. RBM20, a gene for hereditary cardiomyopathy, regulates titin splicing. Nat Med. 2012;18: 766–773. doi: 10.1038/nm.2693 22466703 PMC3569865

[32] Li S, Guo W, Dewey CN, Greaser ML. Rbm20 regulates titin alternative splicing as a splicing repressor. Nucleic Acids Res. 2013;41: 2659–2672. doi: 10.1093/nar/gks1362 23307558 PMC3575840

[33] Kim KK, Nam J, Mukouyama Y-S, Kawamoto S. Rbfox3-regulated alternative splicing of Numb promotes neuronal differentiation during development. J Cell Biol. 2013;200: 443–458. doi: 10.1083/jcb.201206146 23420872 PMC3575530

[34] Bhate A, Parker DJ, Bebee TW, Ahn J, Arif W, Rashan EH, et al. ESRP2 controls an adult splicing programme in hepatocytes to support postnatal liver maturation. Nat Commun. 2015;6: 8768. doi: 10.1038/ncomms9768 26531099 PMC4635967

[35] Ho L, Crabtree GR. Chromatin remodelling during development. Nature. 2010;463: 474–484. doi: 10.1038/nature08911 20110991 PMC3060774

[36] Hota SK, Bruneau BG. ATP-dependent chromatin remodeling during mammalian development. Development. 2016;143: 2882–2897. doi: 10.1242/dev.128892 27531948 PMC5004879

[37] Sokpor G, Xie Y, Rosenbusch J, Tuoc T. Chromatin Remodeling BAF (SWI/SNF) Complexes in Neural Development and Disorders. Frontiers in Molecular Neuroscience. 2017;10. Available: https://www.frontiersin.org/articles/10.3389/fnmol.2017.00243 28824374 10.3389/fnmol.2017.00243 PMC5540894

[38] Cabot B, Cabot RA. Chromatin remodeling in mammalian embryos. Reproduction. 2018;155: R147–R158. doi: 10.1530/REP-17-0488 29339454

[39] Riley CM, Day RL. Central autonomic dysfunction with defective lacrimation; report of five cases. Pediatrics. 1949;3: 468–478. 18118947

[40] Norcliffe-Kaufmann L, Slaugenhaupt SA, Kaufmann H. Familial dysautonomia: History, genotype, phenotype and translational research. Progress in Neurobiology. 2017;152: 131–148. doi: 10.1016/j.pneurobio.2016.06.003 27317387

[41] Jackson MZ, Gruner KA, Qin C, Tourtellotte WG. A neuron autonomous role for the familial dysautonomia gene ELP1 in sympathetic and sensory target tissue innervation. Development. 2014;141: 2452–2461. doi: 10.1242/dev.107797 24917501 PMC4050699

[42] Ibrahim EC, Hims MM, Shomron N, Burge CB, Slaugenhaupt SA, Reed R. Weak definition of IKBKAP exon 20 leads to aberrant splicing in familial dysautonomia. Hum Mutat. 2007;28: 41–53. doi: 10.1002/humu.20401 16964593

[43] Hims MM, Shetty RS, Pickel J, Mull J, Leyne M, Liu L, et al. A humanized IKBKAP transgenic mouse models a tissue-specific human splicing defect. Genomics. 2007;90: 389–396. doi: 10.1016/j.ygeno.2007.05.012 17644305 PMC1976430

[44] Dietrich P, Dragatsis I. Familial Dysautonomia: Mechanisms and Models. Genet Mol Biol. 2016;39: 497–514. doi: 10.1590/1678-4685-GMB-2015-0335 27561110 PMC5127153

[45] Sinha R, Kim YJ, Nomakuchi T, Sahashi K, Hua Y, Rigo F, et al. Antisense oligonucleotides correct the familial dysautonomia splicing defect in IKBKAP transgenic mice. Nucleic Acids Res. 2018;46: 4833–4844. doi: 10.1093/nar/gky249 29672717 PMC6007753

[46] Donadon I, Pinotti M, Rajkowska K, Pianigiani G, Barbon E, Morini E, et al. Exon-specific U1 snRNAs improve ELP1 exon 20 definition and rescue ELP1 protein expression in a familial dysautonomia mouse model. Human Molecular Genetics. 2018;27: 2466–2476. doi: 10.1093/hmg/ddy151 29701768 PMC6030917

[47] Romano G, Riccardi F, Bussani E, Vodret S, Licastro D, Ragone I, et al. Rescue of a familial dysautonomia mouse model by AAV9-Exon-specific U1 snRNA. The American Journal of Human Genetics. 2022;109: 1534–1548. doi: 10.1016/j.ajhg.2022.07.004 35905737 PMC9388384

[48] Ajiro M, Awaya T, Kim YJ, Iida K, Denawa M, Tanaka N, et al. Therapeutic manipulation of IKBKAP mis-splicing with a small molecule to cure familial dysautonomia. Nat Commun. 2021;12: 4507. doi: 10.1038/s41467-021-24705-5 34301951 PMC8302731

[49] Gao D, Morini E, Salani M, Krauson AJ, Chekuri A, Sharma N, et al. A deep learning approach to identify gene targets of a therapeutic for human splicing disorders. Nat Commun. 2021;12: 3332. doi: 10.1038/s41467-021-23663-2 34099697 PMC8185002

[50] Morini E, Gao D, Montgomery CM, Salani M, Mazzasette C, Krussig TA, et al. ELP1 Splicing Correction Reverses Proprioceptive Sensory Loss in Familial Dysautonomia. Am J Hum Genet. 2019;104: 638–650. doi: 10.1016/j.ajhg.2019.02.009 30905397 PMC6451698

[51] Slaugenhaupt SA, Mull J, Leyne M, Cuajungco MP, Gill SP, Hims MM, et al. Rescue of a human mRNA splicing defect by the plant cytokinin kinetin. Hum Mol Genet. 2004;13: 429–436. doi: 10.1093/hmg/ddh046 14709595

[52] Singh J, Padgett RA. Rates of in situ transcription and splicing in large human genes. Nat Struct Mol Biol. 2009;16: 1128–1133. doi: 10.1038/nsmb.1666 19820712 PMC2783620

[53] Dobin A, Gingeras TR. Mapping RNA-seq Reads with STAR. Curr Protoc Bioinformatics. 2015;51: 11.14.1–11.14.19. doi: 10.1002/0471250953.bi1114s51 26334920 PMC4631051

[54] Love MI, Huber W, Anders S. Moderated estimation of fold change and dispersion for RNA-seq data with DESeq2. Genome Biol. 2014;15: 550. doi: 10.1186/s13059-014-0550-8 25516281 PMC4302049

[55] Shen S, Park JW, Lu Z, Lin L, Henry MD, Wu YN, et al. rMATS: robust and flexible detection of differential alternative splicing from replicate RNA-Seq data. Proc Natl Acad Sci U S A. 2014;111: E5593–5601. doi: 10.1073/pnas.1419161111 25480548 PMC4280593

[56] Irimia M, Weatheritt RJ, Ellis JD, Parikshak NN, Gonatopoulos-Pournatzis T, Babor M, et al. A highly conserved program of neuronal microexons is misregulated in autistic brains. Cell. 2014;159: 1511–1523. doi: 10.1016/j.cell.2014.11.035 25525873 PMC4390143

[57] Capranico G, Marinello J, Baranello L. Dissecting the transcriptional functions of human DNA topoisomerase I by selective inhibitors: implications for physiological and therapeutic modulation of enzyme activity. Biochim Biophys Acta. 2010;1806: 240–250. doi: 10.1016/j.bbcan.2010.06.003 20600630

[58] Ip JY, Schmidt D, Pan Q, Ramani AK, Fraser AG, Odom DT, et al. Global impact of RNA polymerase II elongation inhibition on alternative splicing regulation. Genome Research. 2011;21: 390–401. doi: 10.1101/gr.111070.110 21163941 PMC3044853

[59] C Quaresma AJ, Bugai A, Barboric M. Cracking the control of RNA polymerase II elongation by 7SK snRNP and P-TEFb. Nucleic Acids Res. 2016;44: 7527–7539. doi: 10.1093/nar/gkw585 27369380 PMC5027500

[60] Weaver ICG, Korgan AC, Lee K, Wheeler RV, Hundert AS, Goguen D. Stress and the Emerging Roles of Chromatin Remodeling in Signal Integration and Stable Transmission of Reversible Phenotypes. Front Behav Neurosci. 2017;11. doi: 10.3389/fnbeh.2017.00041 28360846 PMC5350110

[61] Tie F, Banerjee R, Stratton CA, Prasad-Sinha J, Stepanik V, Zlobin A, et al. CBP-mediated acetylation of histone H3 lysine 27 antagonizes Drosophila Polycomb silencing. Development. 2009;136: 3131–3141. doi: 10.1242/dev.037127 19700617 PMC2730368

[62] Pasini D, Malatesta M, Jung HR, Walfridsson J, Willer A, Olsson L, et al. Characterization of an antagonistic switch between histone H3 lysine 27 methylation and acetylation in the transcriptional regulation of Polycomb group target genes. Nucleic Acids Res. 2010;38: 4958–4969. doi: 10.1093/nar/gkq244 20385584 PMC2926606

[63] Qi W, Zhao K, Gu J, Huang Y, Wang Y, Zhang H, et al. An allosteric PRC2 inhibitor targeting the H3K27me3 binding pocket of EED. Nat Chem Biol. 2017;13: 381–388. doi: 10.1038/nchembio.2304 28135235

[64] Lavarone E, Barbieri CM, Pasini D. Dissecting the role of H3K27 acetylation and methylation in PRC2 mediated control of cellular identity. Nat Commun. 2019;10: 1679. doi: 10.1038/s41467-019-09624-w 30976011 PMC6459869

[65] Buratti E, Dörk T, Zuccato E, Pagani F, Romano M, Baralle FE. Nuclear factor TDP-43 and SR proteins promote in vitro and in vivo CFTR exon 9 skipping. EMBO J. 2001;20: 1774–1784. doi: 10.1093/emboj/20.7.1774 11285240 PMC145463

[66] Tajnik M, Rogalska ME, Bussani E, Barbon E, Balestra D, Pinotti M, et al. Molecular Basis and Therapeutic Strategies to Rescue Factor IX Variants That Affect Splicing and Protein Function. PLoS Genet. 2016;12: e1006082. doi: 10.1371/journal.pgen.1006082 27227676 PMC4882169

[67] Brykczynska U, Hisano M, Erkek S, Ramos L, Oakeley EJ, Roloff TC, et al. Repressive and active histone methylation mark distinct promoters in human and mouse spermatozoa. Nat Struct Mol Biol. 2010;17: 679–687. doi: 10.1038/nsmb.1821 20473313

[68] Saxena M, Roman AKS, O’Neill NK, Sulahian R, Jadhav U, Shivdasani RA. Transcription factor-dependent ‘anti-repressive’ mammalian enhancers exclude H3K27me3 from extended genomic domains. Genes Dev. 2017;31: 2391–2404. doi: 10.1101/gad.308536.117 29321178 PMC5795785

[69] Jadhav U, Manieri E, Nalapareddy K, Madha S, Chakrabarti S, Wucherpfennig K, et al. Replicational Dilution of H3K27me3 in Mammalian Cells and the Role of Poised Promoters. Molecular Cell. 2020;78: 141–151.e5. doi: 10.1016/j.molcel.2020.01.017 32027840 PMC7376365

[70] Agirre E, Oldfield AJ, Bellora N, Segelle A, Luco RF. Splicing-associated chromatin signatures: a combinatorial and position-dependent role for histone marks in splicing definition. Nat Commun. 2021;12: 682. doi: 10.1038/s41467-021-20979-x 33514745 PMC7846797

[71] Xu Y, Zhao W, Olson SD, Prabhakara KS, Zhou X. Alternative splicing links histone modifications to stem cell fate decision. Genome Biol. 2018;19: 133. doi: 10.1186/s13059-018-1512-3 30217220 PMC6138936

[72] Podlaha O, De S, Gonen M, Michor F. Histone modifications are associated with transcript isoform diversity in normal and cancer cells. PLoS Comput Biol. 2014;10: e1003611. doi: 10.1371/journal.pcbi.1003611 24901363 PMC4046914

[73] Bruun GH, Bang JMV, Christensen LL, Brøner S, Petersen USS, Guerra B, et al. Blocking of an intronic splicing silencer completely rescues IKBKAP exon 20 splicing in familial dysautonomia patient cells. Nucleic Acids Research. 2018;46: 7938–7952. doi: 10.1093/nar/gky395 29762696 PMC6125618

[74] Bracken AP, Helin K. Polycomb group proteins: navigators of lineage pathways led astray in cancer. Nat Rev Cancer. 2009;9: 773–784. doi: 10.1038/nrc2736 19851313

[75] Kim J, Kim H. Recruitment and biological consequences of histone modification of H3K27me3 and H3K9me3. ILAR J. 2012;53: 232–239. doi: 10.1093/ilar.53.3-4.232 23744963 PMC3747788

[76] Haithcock E, Dayani Y, Neufeld E, Zahand AJ, Feinstein N, Mattout A, et al. Age-related changes of nuclear architecture in Caenorhabditis elegans. Proc Natl Acad Sci U S A. 2005;102: 16690–16695. doi: 10.1073/pnas.0506955102 16269543 PMC1283819

[77] Scaffidi P, Misteli T. Lamin A-dependent nuclear defects in human aging. Science. 2006;312: 1059–1063. doi: 10.1126/science.1127168 16645051 PMC1855250

[78] Brandt A, Krohne G, Grosshans J. The farnesylated nuclear proteins KUGELKERN and LAMIN B promote aging-like phenotypes in Drosophila flies. Aging Cell. 2008;7: 541–551. doi: 10.1111/j.1474-9726.2008.00406.x 18494863

[79] Schwörer S, Becker F, Feller C, Baig AH, Köber U, Henze H, et al. Epigenetic stress responses induce muscle stem-cell ageing by Hoxa9 developmental signals. Nature. 2016;540: 428–432. doi: 10.1038/nature20603 27919074 PMC5415306

[80] Lee TI, Jenner RG, Boyer LA, Guenther MG, Levine SS, Kumar RM, et al. Control of developmental regulators by Polycomb in human embryonic stem cells. Cell. 2006;125: 301–313. doi: 10.1016/j.cell.2006.02.043 16630818 PMC3773330

[81] Cheung P, Vallania F, Warsinske HC, Donato M, Schaffert S, Chang SE, et al. Single-Cell Chromatin Modification Profiling Reveals Increased Epigenetic Variations with Aging. Cell. 2018;173: 1385–1397.e14. doi: 10.1016/j.cell.2018.03.079 29706550 PMC5984186

[82] Liu X, Bushnell DA, Kornberg RD. RNA polymerase II transcription: Structure and mechanism. Biochimica et Biophysica Acta (BBA)—Gene Regulatory Mechanisms. 2013;1829: 2–8. doi: 10.1016/j.bbagrm.2012.09.003 23000482 PMC4244541

[83] Noer A, Lindeman LC, Collas P. Histone H3 modifications associated with differentiation and long-term culture of mesenchymal adipose stem cells. Stem Cells Dev. 2009;18: 725–736. doi: 10.1089/scd.2008.0189 18771397

[84] Baumgart M, Groth M, Priebe S, Savino A, Testa G, Dix A, et al. RNA-seq of the aging brain in the short-lived fish N. furzeri—conserved pathways and novel genes associated with neurogenesis. Aging Cell. 2014;13: 965–974. doi: 10.1111/acel.12257 25059688 PMC4326923

[85] Cencioni C, Heid J, Krepelova A, Rasa SMM, Kuenne C, Guenther S, et al. Aging Triggers H3K27 Trimethylation Hoarding in the Chromatin of Nothobranchius furzeri Skeletal Muscle. Cells. 2019;8: E1169. doi: 10.3390/cells8101169 31569376 PMC6829443

[86] Wang CM, Tsai SN, Yew TW, Kwan YW, Ngai SM. Identification of histone methylation multiplicities patterns in the brain of senescence-accelerated prone mouse 8. Biogerontology. 2010;11: 87–102. doi: 10.1007/s10522-009-9231-5 19434510

[87] Siebold AP, Banerjee R, Tie F, Kiss DL, Moskowitz J, Harte PJ. Polycomb Repressive Complex 2 and Trithorax modulate Drosophila longevity and stress resistance. Proc Natl Acad Sci U S A. 2010;107: 169–174. doi: 10.1073/pnas.0907739107 20018689 PMC2806727

[88] Maures TJ, Greer EL, Hauswirth AG, Brunet A. The H3K27 demethylase UTX-1 regulates C. elegans lifespan in a germline-independent, insulin-dependent manner. Aging Cell. 2011;10: 980–990. doi: 10.1111/j.1474-9726.2011.00738.x 21834846 PMC3215905

[89] Kosti A, de Araujo PR, Li W-Q, Guardia GDA, Chiou J, Yi C, et al. The RNA-binding protein SERBP1 functions as a novel oncogenic factor in glioblastoma by bridging cancer metabolism and epigenetic regulation. Genome Biol. 2020;21: 195. doi: 10.1186/s13059-020-02115-y 32762776 PMC7412812

[90] Duan R, Du W, Guo W. EZH2: a novel target for cancer treatment. J Hematol Oncol. 2020;13: 104. doi: 10.1186/s13045-020-00937-8 32723346 PMC7385862

[91] Luco RF, Allo M, Schor IE, Kornblihtt AR, Misteli T. Epigenetics in Alternative Pre-mRNA Splicing. Cell. 2011;144: 16–26. doi: 10.1016/j.cell.2010.11.056 21215366 PMC3038581

