# Supplementary figures and images for: Age-dependent regulation of ELP1 exon 20 splicing in Familial Dysautonomia by RNA Polymerase II kinetics and chromatin structure
Source: PLoS One. 2024 Jun 3;19(6):e0298965. doi: 10.1371/journal.pone.0298965 (PMC11146744; doi:10.1371/journal.pone.0298965)

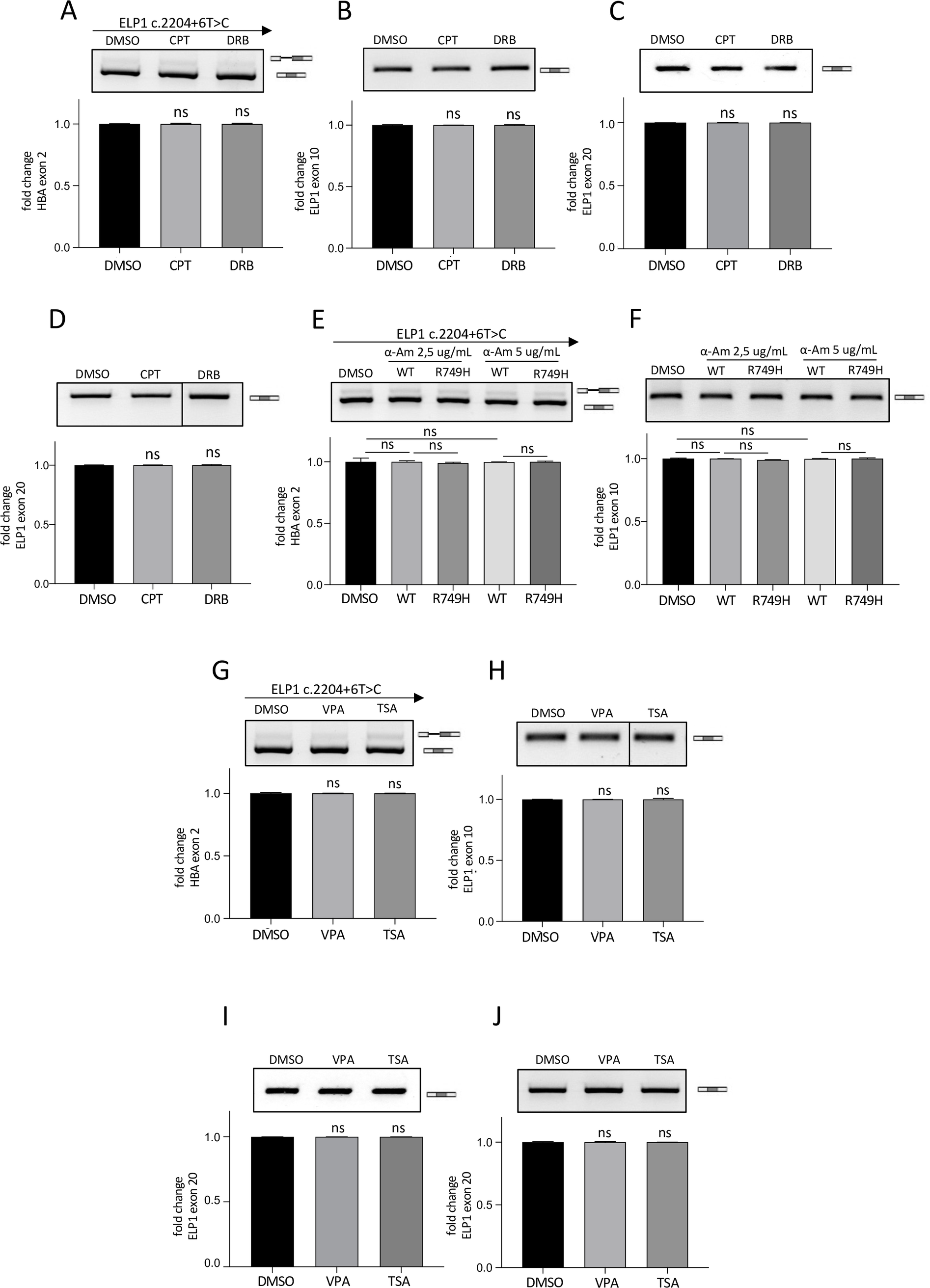

Supplement: S1 Fig — (A) HEK 293T cells were transfected with ELP1 mutant minigene (c.2204+6T>C) and treated with 0.1% DMSO or CPT 12 uM or DRB 75 uM for 24 h. DMSO-treated cells are set to 1. (B) HEK 293T cells were treated with 0.1% DMSO or CPT 12 uM or DRB 75 uM for 24 h. DMSO-treated cells are set to 1. (C) HEK 293T cells were treated with 0.1% DMSO or CPT 12 uM or DRB 75 uM for 24 h. DMSO-treated cells are set to 1. (D) Healthy donor (WT) fibroblasts were treated with 0.1% DMSO or CPT 12 uM or DRB 20 uM for 24 h. DMSO-treated cells are set to 1. (E) HEK 293T cells were co-transfected with ELP1 mutant minigene and the expression vectors for WTresPol II and R749H slow mutant RNA Polymerase II followed by the addition of 2.5 ug/mL or 5 ug/mL of α-amanitin for 24h. Cells transfected only with ELP1 mutant minigene and treated with 0.1% DMSO are set to 1. (F) HEK 293T cells were co-transfected with the expression vectors for WTresPol II and R749H slow mutant RNAPII followed by the addition of 2.5 ug/mL or 5 ug/mL of α-amanitin for 24h. DMSO-treated are set to 1. (G) HEK 293T cells were transfected with ELP1 mutant minigene (c.2204+6T>C) and treated with 0.1% DMSO or VPA 4 mM or TSA 1 ug/mL for 24 h. DMSO-treated cells are set to 1. (H) HEK 293T cells were treated with 0.1% DMSO or VPA 4 mM or TSA 1 ug/mL for 24 h. DMSO-treated cells are set to 1. (I) HEK 293T cells were treated with 0.1% DMSO or VPA 4 mM or TSA 1 ug/mL for 24 h. DMSO-treated cells are set to 1. (J) Healthy donor (WT) fibroblasts were treated with 0.1% DMSO or VPA 8 mM or TSA 1 ug/mL for 24 h. DMSO-treated cells are set to 1. In all panels, HBA exon 2, ELP1 exon 10 and ELP1 exon 20 inclusion bands are indicated and in panels (A), (E) and (G) the HBA upper band corresponds to intron 1 retention. The intensity of the bands was measured with ImageJ software and histogram below gel displays the percentage of exon inclusion expressed as fold change. Dividing lines indicate cropping and annealing of the same agarose gel or expe [file pone.0298965.s001.tif]

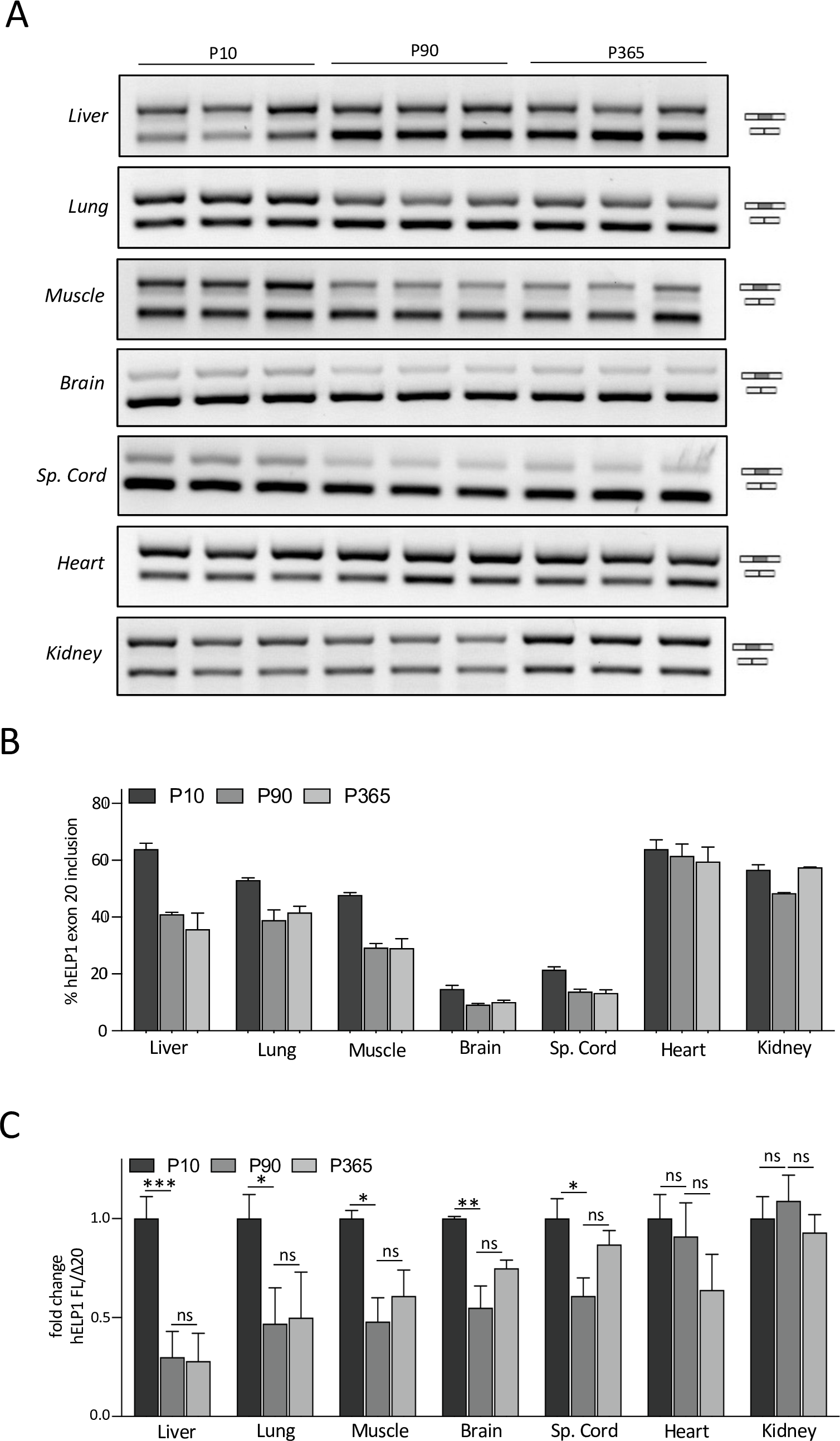

Supplement: S2 Fig — (A) Endpoint PCR of hELP1 splicing pattern in FD mouse sacrificed at P10, P90 and P365. Identity of exon inclusion (202 bp) and skipping (128 bp) bands are indicated on the right and tissues analyzed on the left of the gel. (B) Quantification of the intensity of ePCR gels bands with ImageJ software. Data are expressed as percentage of hELP1 exon 20 inclusion. (C) SYBR green based-qPCR quantification of the ratio of hELP1 FL (mRNAs including exon 20) to Δ20 (mRNAs lacking exon 20) transcripts in FD mouse model sacrificed at P10, P90 and P365. In each tissue, the expression level of P10 animals is set to 1. Data are expressed as mean + s.e.m. of 3 mice for each age. Statistical analysis was performed using OneWay ANOVA (ns: not significant; * p<0.05; ** p<0.01; ***p<0.001). (TIF) [file pone.0298965.s002.tif]

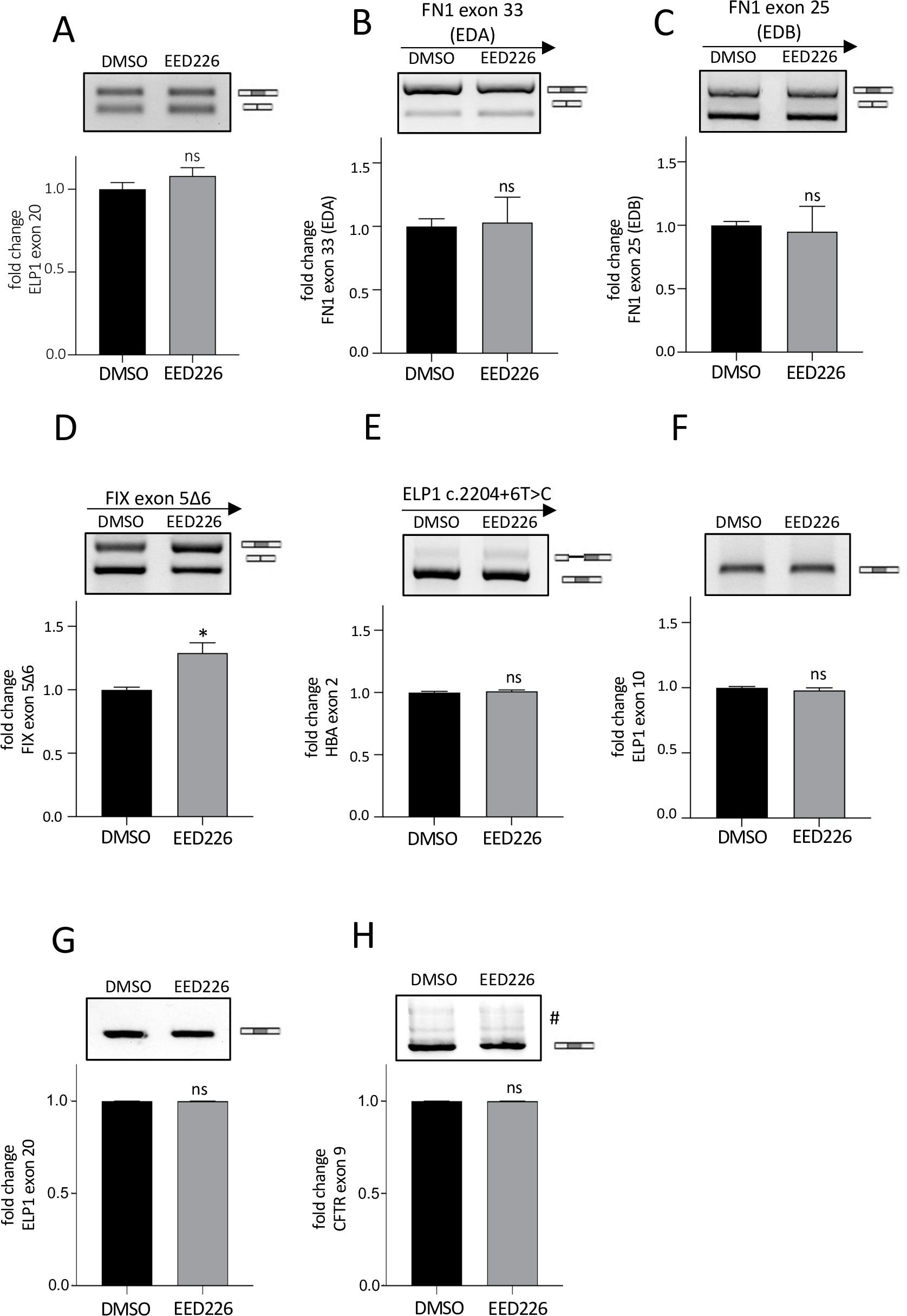

Supplement: S3 Fig — (A) FD patients’ fibroblasts were treated with 0.1% DMSO or EED226 100 uM for 24 h. DMSO-treated cells are set to 1. HEK 293T cells were transfected with (B) FN1 exon 33 (EDA) minigene, (C) FN1 exon 25 (EDB) minigene and (D) FIX exon 5Δ6 minigene and treated with 0.1% DMSO or EED226 100 uM for 24 h. DMSO-treated cells are set to 1. (E) HEK 293T cells were transfected with ELP1 mutant minigene (c.2204+6T>C) and treated with 0.1% DMSO or EED226 100 uM for 24 h. DMSO-treated cells are set to 1. (F) HEK 293T cells were treated with 0.1% DMSO or EED226 100 uM for 24 h. DMSO-treated cells are set to 1. (G) HEK 293T cells were treated with 0.1% DMSO or EED226 100 uM for 24 h. DMSO-treated cells are set to 1. (H) HEK 293T cells were transfected with CFTR exon 9 wt minigene and treated with 0.1% DMSO or EED226 100 uM for 24 h. DMSO-treated cells are set to 1. # denotes PCR artifacts. In panel (B), FN1 EDA inclusion and exclusion bands are indicated. In panel (C), FN1 EDB inclusion and exclusion bands are indicated. In panel (D), FIX exon 5Δ6 inclusion and exclusion bands are indicated. In panel (E), HBA exon 2 inclusion band is indicated and the HBA upper band corresponds to intron 1 retention. In panels (F), (G) and (H) ELP1 exon 10, ELP1 exon 20 wt and CFTR exon 9 wt inclusion bands are indicated, respectively. In all panels, the intensity of the bands was measured with ImageJ software and histogram below gel displays the percentage of exon inclusion expressed as fold change. Data are expressed as mean + S.D. of n = 3 experiments in triplicate. Statistical analysis was performed using Student t-test (ns: not significant; * p< 0.05). (TIF) [file pone.0298965.s003.tif]

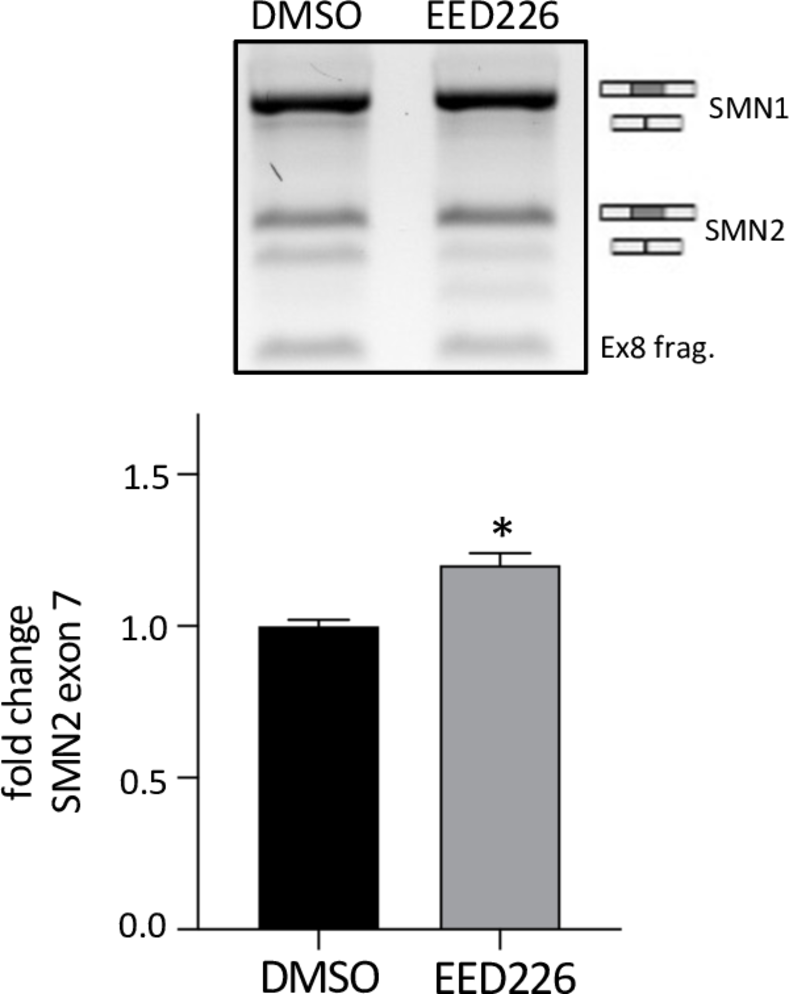

Supplement: S4 Fig — HEK 293T cells were treated with 0.1% DMSO or EED226 100uM for 48 h and RT-PCR amplified fragments digested with DdeI restriction enzyme to obtain SMN1 and SMN2 exon 7 inclusion (FL) and exclusion (Δ7) fragments, respectively. The SMN2 exon 7 inclusion and exclusion bands and a fragment of exon 8 are indicated. DMSO-treated cells are set to 1. The intensity of the bands was measured with ImageJ software and histogram below gel displays the percentage of exon inclusion expressed as fold change. Data are expressed as mean + S.D. of n = 3 experiments in triplicate. Statistical analysis was performed using Student t-test (* p<0.05). (TIF) [file pone.0298965.s004.tif]
