# Supplementary material for: Age-dependent regulation of ELP1 exon 20 splicing in Familial Dysautonomia by RNA Polymerase II kinetics and chromatin structure
Source: PLoS One. 2024 Jun 3;19(6):e0298965. doi: 10.1371/journal.pone.0298965 (PMC11146744; doi:10.1371/journal.pone.0298965)
Supplement: S1 Table — (PDF) [file pone.0298965.s005.pdf]

[redacted]		
ELP1 exon 20	CAACTTCAAGCTCCTAAGCCACTGC	GATTCTCAGCTTTCTCATGCATTC
HBA exon 2	ACGCTGGCGAGTATGGTG	GCAGGTCACCAGCAGGCAGT
SMN2 exon 7	ACTCACTATAGGCTAGCCT	CTGTCTAGACCTTACACTTCGCAATGTCCATTCATGAA
CFTR exon 9 TG11T5 / wt	CAACTTCAAGCTCCTAAGCCACTGC	GTCACCAGGAAGTTGGTTAAATCA
FN1 exon 33 (EDA)	CAACTTCAAGCTCCTAAGCCACTGC	CTCGATATCCAGTGAGCTGAACATTGG
FN1 exon 25 (EDB)	CAACTTCAAGCTCCTAAGCCACTGC	GTCACCAGGAAGTTGGTTAAATCA
FIX exon 5Δ6	CAACTTCAAGCTCCTAAGCCACTGC	GTCACCAGGAAGTTGGTTAAATCA
[redacted]	[redacted]	[redacted]
ELP1 intron 9/exon 10	GACAGATCTTTTTTCCCTTCTAGG	GCTGGGGTACTACAGCTGTCTT
ELP1 exon 20/ intron 20	TCATCGAGCCCTGGTTTTAGC	ACAAGCTAACTAGTCGCAAAC
SMN exon 2/intron 2	TGATAAAAGCATATGATAAAGC	TGAAAACTGAATAACATGTAC
SMN intron 7/exon 8	ATAGCTATCTATGTCTATATAGC	GGAATGTGAGCACCTTCCTTC
[redacted]	[redacted]	[redacted]
ELP1 exon 10	GATAAACCCAACCAGCAGGA	TTCCAACAGTCCAGAGCTGA
SMN2 exon 7	ATAATTCCCCCACCACCTCCC	TTGCCACATACGCCTCACATAC
[redacted]	[redacted]	[redacted]
hELP1 exon 10	GACAGATCTTTTTTCCCTTCTAGG	GCTGGGGTACTACAGCTGTCTT
hELP1 exon 20	AATTTATTTAAGATGCCAAGGGGAA	ACAATGGCGCTTACTTGTCC
hELP1 exon 29	CTGCTGCTTATTGTCTCTACAGG	AAGGGAGGAATTGAGTTTACCTG
[redacted]	[redacted]	[redacted]
hELP1 exon 20	GGCCGGCCTGAGCAGCAATCATGTGTCC	GATTCTCAGCTTTCTCATGCATTC
hELP1 FL mRNA	GCAGCAATCATGTGTCCCA	ACCAGGGCTCGATGATGAA
hELP1 Δ20 mRNA	CACAAAGCTTGTATTACAGACT	GAAGGTTTCCACATTTCCAAG
hELP1 total mRNA	GCTGTTCCCACACCCTGT	AGGGTCAGCACTTGGACAA
Mouse GAPDH	ATGGTGAAGGTCGGTGTGAA	GTTGATGGCAACAATCTCCA
Human U6 snRNA	GCTTCGGCAGCACATATACTAAAAT	ACGAATTTGCGTGTCATCCTT

S1 Table: Oligonucleotides used in this study.
