# Supplementary material for: Age-dependent regulation of ELP1 exon 20 splicing in Familial Dysautonomia by RNA Polymerase II kinetics and chromatin structure
Source: PLoS One. 2024 Jun 3;19(6):e0298965. doi: 10.1371/journal.pone.0298965 (PMC11146744; doi:10.1371/journal.pone.0298965)
Supplement: S2 Table — (PDF) [file pone.0298965.s006.pdf]

Group	Alternative Splicing events	N° of analyzed events	Significant different events		
			%	N° tot	Up/down
EED226 vs control cells	SE	22075	3,30	728	408/320
	MXE	2637	6,86	181	83/98
	RI	2354	10,75	253	79/174
	A3SS	1656	5,4	90	46/44
	A5SS	1155	4,7	55	25/30

S2 Table: Summary table of alternative splicing analysis in HEK 293T cells treated with EED226. The total number of analyzed events and the percentage of the significant ones are reported for each category (SE = skipped exon; MXE = mutually exclusive exon; RI = retained intron; A3SS = alternative 3' splice site; A5SS = alternative 5' splice site). Number of analyzed events = sum (Σ) of the counts means ≥ 20 (MXE-SE-RI) or ≥ 40 (A3SS-A5SS). Significant events: FDR ≤ 0.5 and Inclusion Level Difference ≤ -0.05 or ≥ 0.05.
