# Supplementary material for: Age-dependent regulation of ELP1 exon 20 splicing in Familial Dysautonomia by RNA Polymerase II kinetics and chromatin structure
Source: PLoS One. 2024 Jun 3;19(6):e0298965. doi: 10.1371/journal.pone.0298965 (PMC11146744; doi:10.1371/journal.pone.0298965)

Supplementary information file

Age-dependent regulation of ELP1 exon 20 splicing in Familial Dysautonomia by RNA Polymerase II kinetics and chromatin structure

Federico Riccardi[1], Giulia Romano[1], Danilo Licastro[2] and Franco Pagani[1]*

[1]Human Molecular Genetics, International Centre for Genetic Engineering and Biotechnology, Padriciano, Trieste, Italy

[2]Laboratorio di Genomica ed Epigenomica, AREA Science Park, Padriciano, Trieste, Italy

This file includes all the original uncropped and unadjusted images underlying all blot or gel results showed in the paper. In S1 panel J, we removed the line between DMSO-VPA lanes and TSA lane as the starting image was the same. We sincerely apologize for the inconvenience. Details on electrophoreses are reported in the manuscript.

Fig. 1

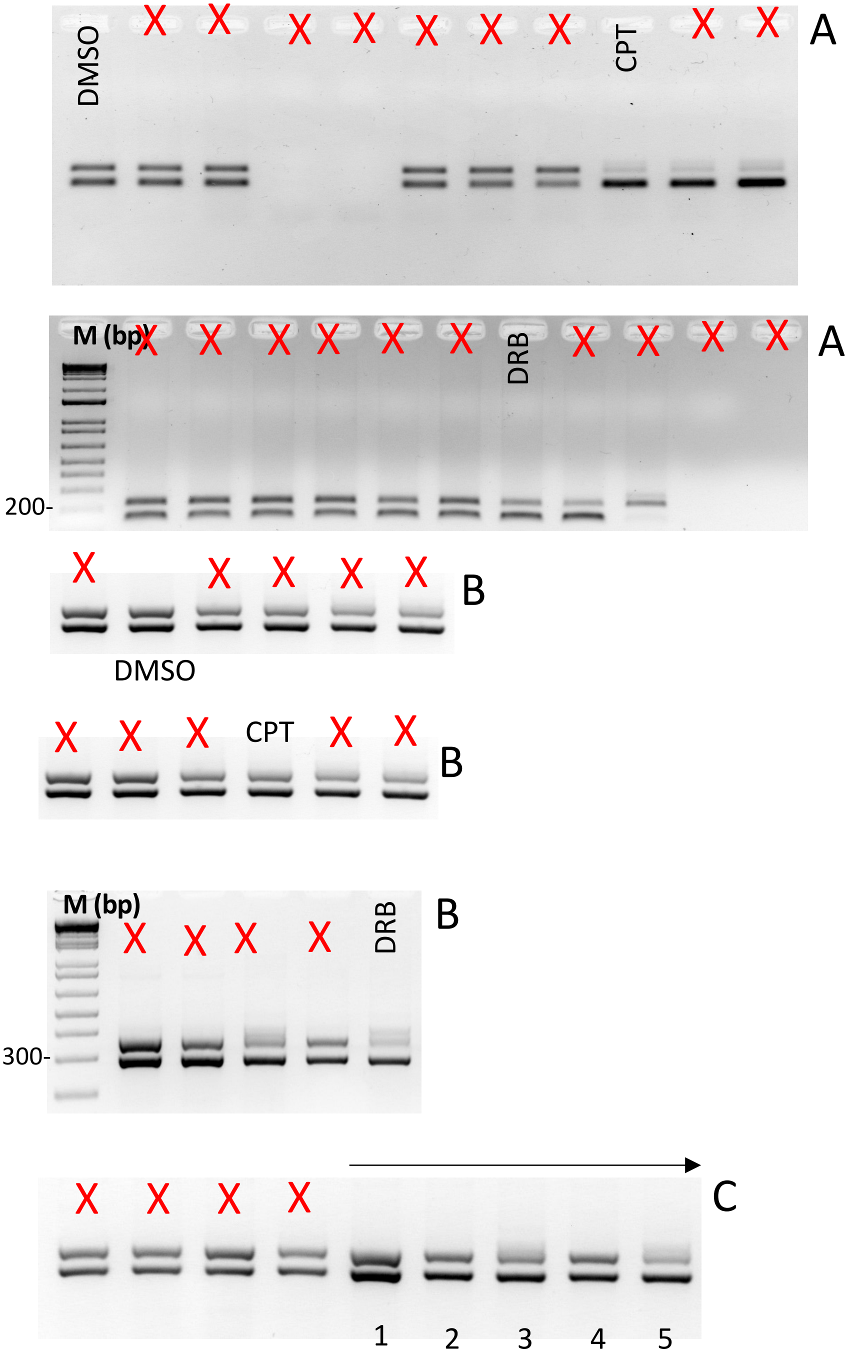

Fig. 1

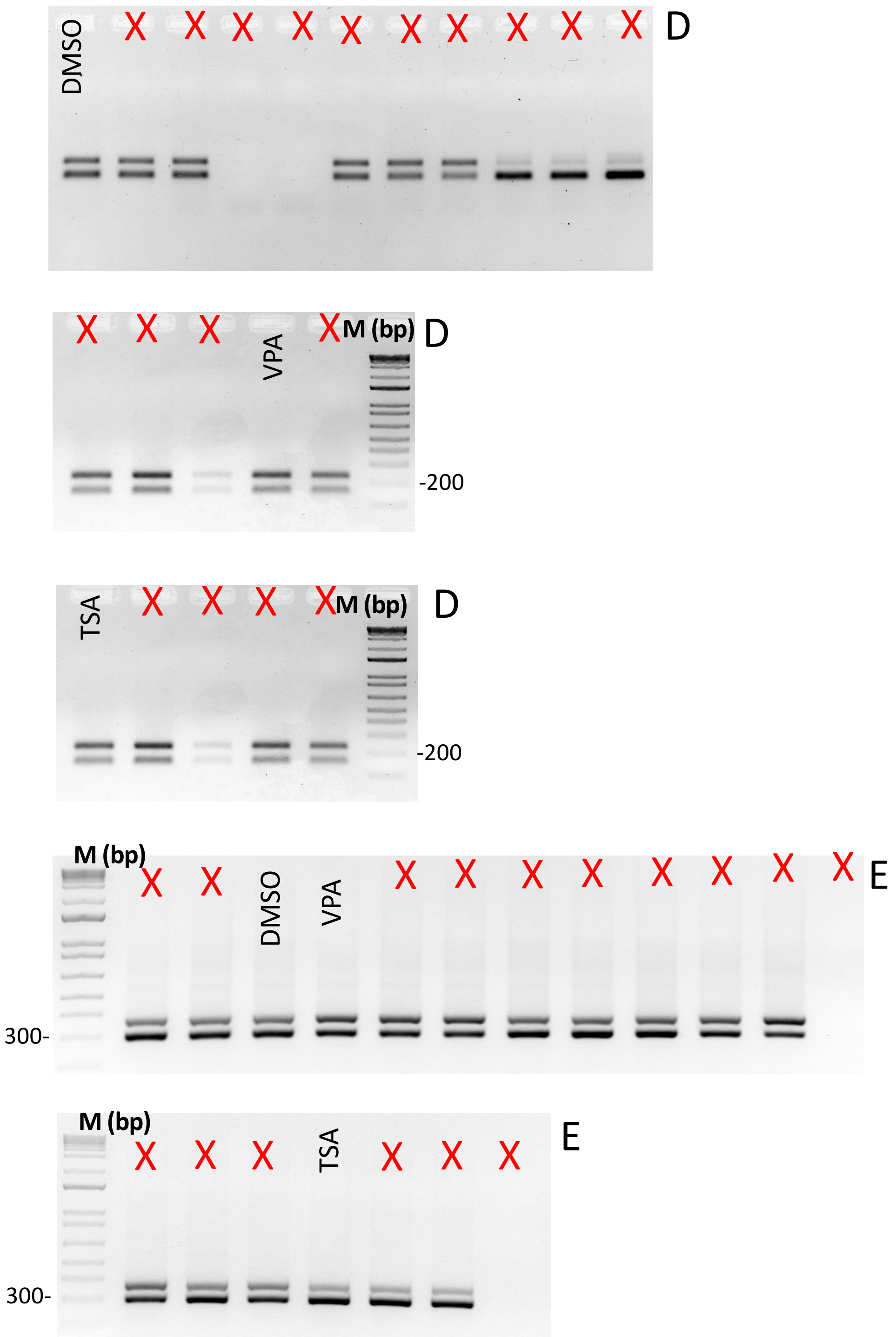

Fig. 3 C-D

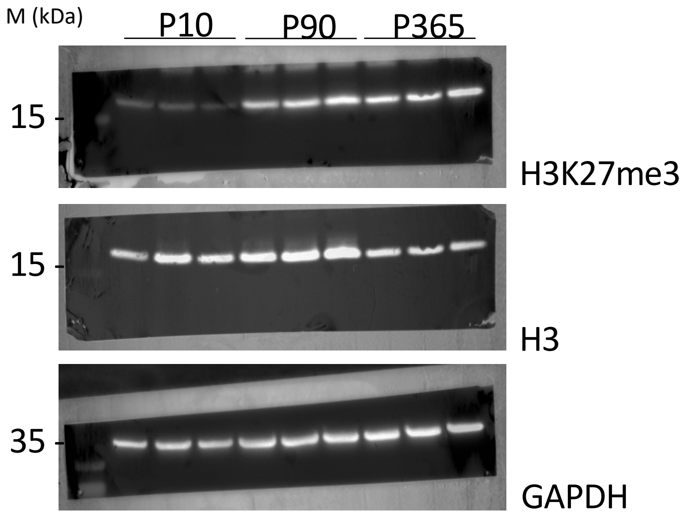
M (kDa)
P10
P90
P365
15
H3K27me3
15
H3
35
GAPDH

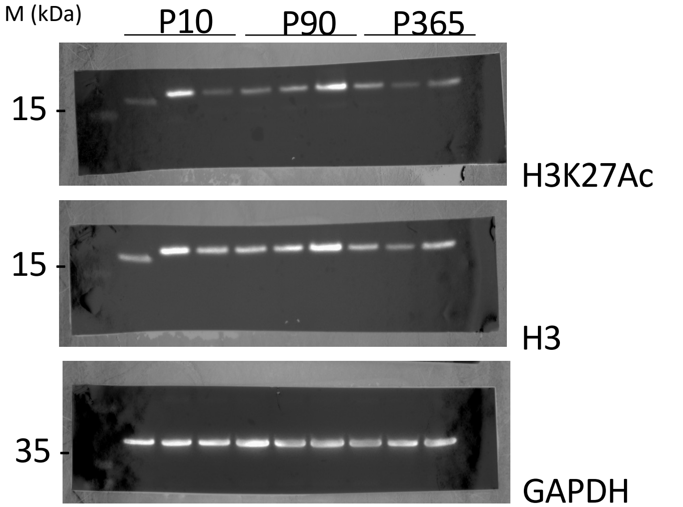
M (kDa)
P10
P90
P365
15
H3K27Ac
15
H3
35
GAPDH

Fig. 4 A-B

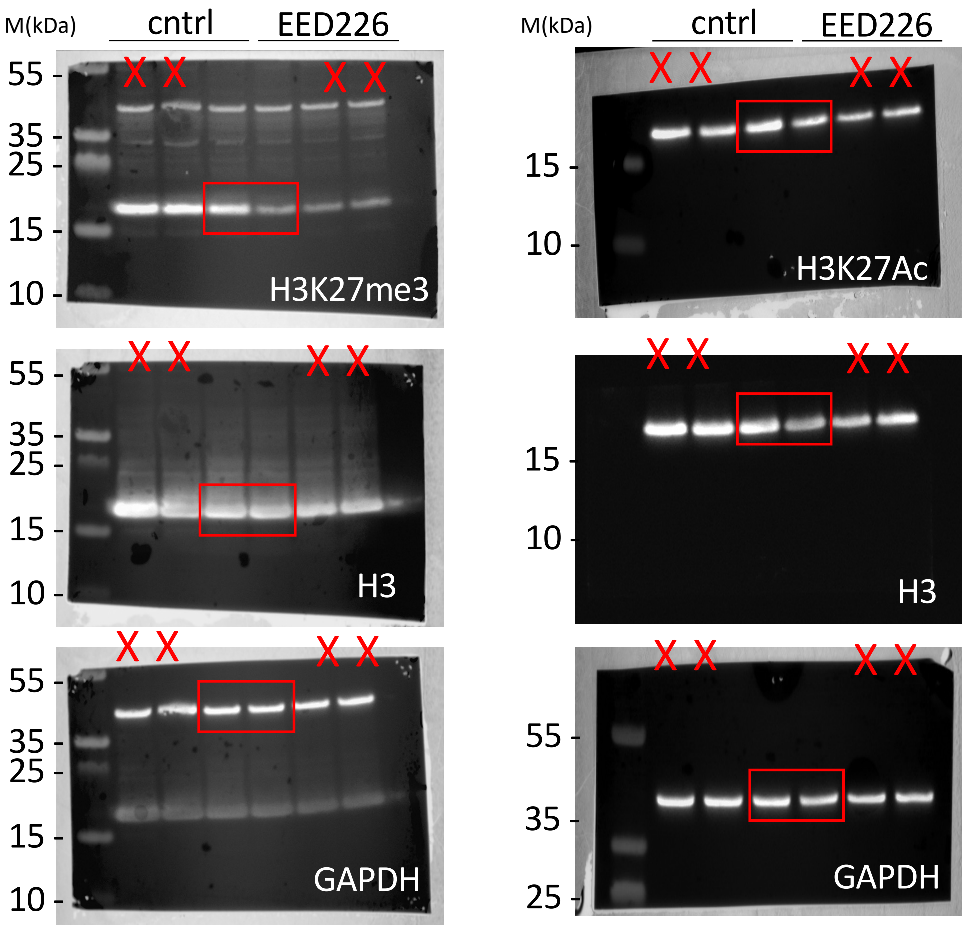
M(kDa)
cntrl
EED226
55
35
25
15
10
H3K27me3
H3
GAPDH
M(kDa)
cntrl
EED226
15
10
H3K27Ac
15
10
H3
55
35
25
GAPDH

Fig. 4 C

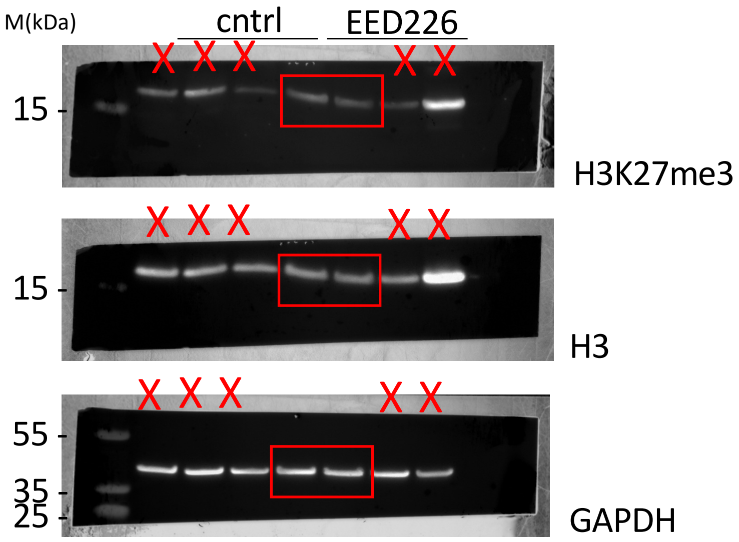

Fig. 4

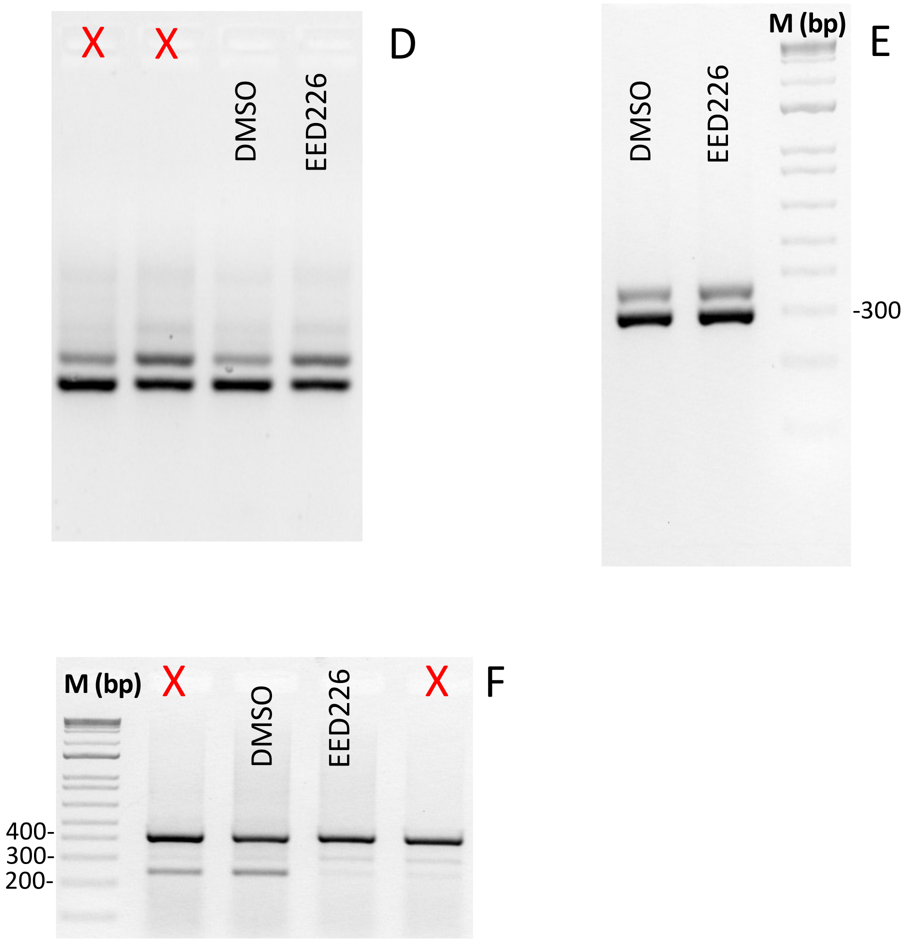

X
X
DMSO
EED226
D
M (bp)
DMSO
EED226
E
-300
M (bp)
X
DMSO
EED226
X
F
400-
300-
200-

Supp Fig 1

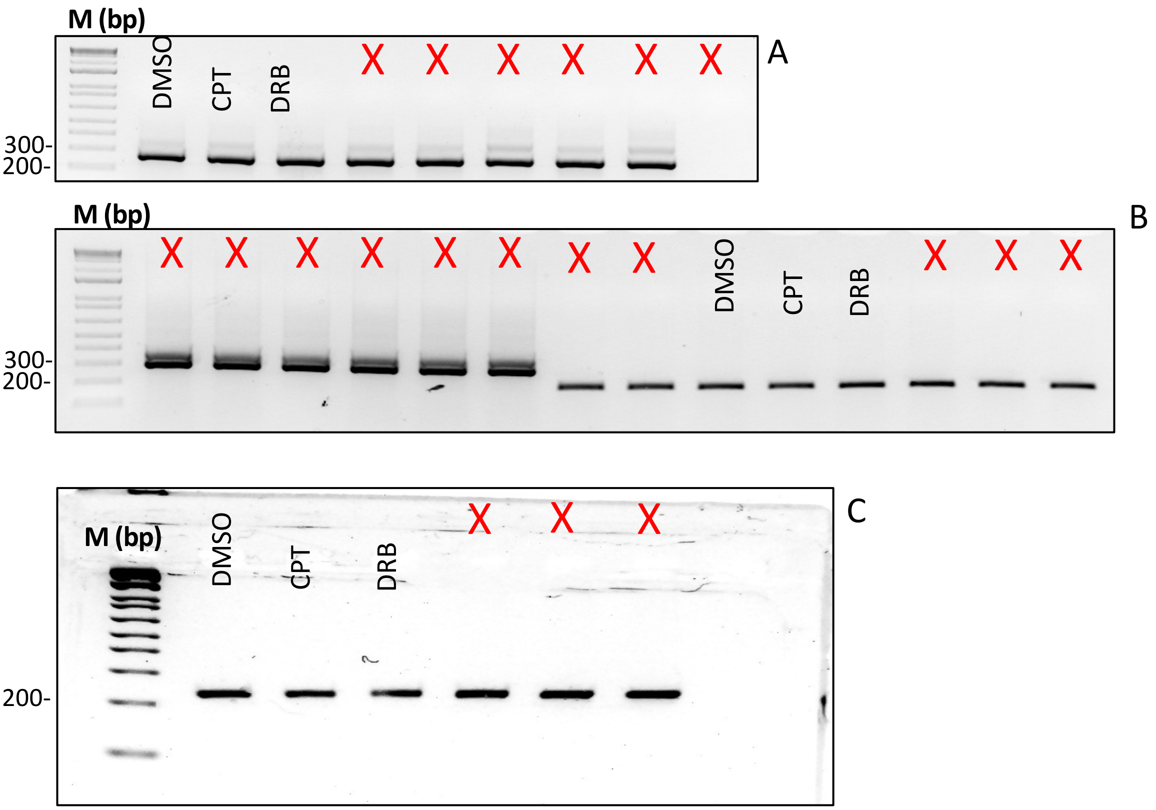

M (bp)
DMSO
CPT
DRB
X
X
X
X
X
X
300-
200-
A
M (bp)
X
X
X
X
X
X
X
X
DMSO
CPT
DRB
X
X
X
300-
200-
B
M (bp)
DMSO
CPT
DRB
X
X
X
200-
C

Supp Fig 1

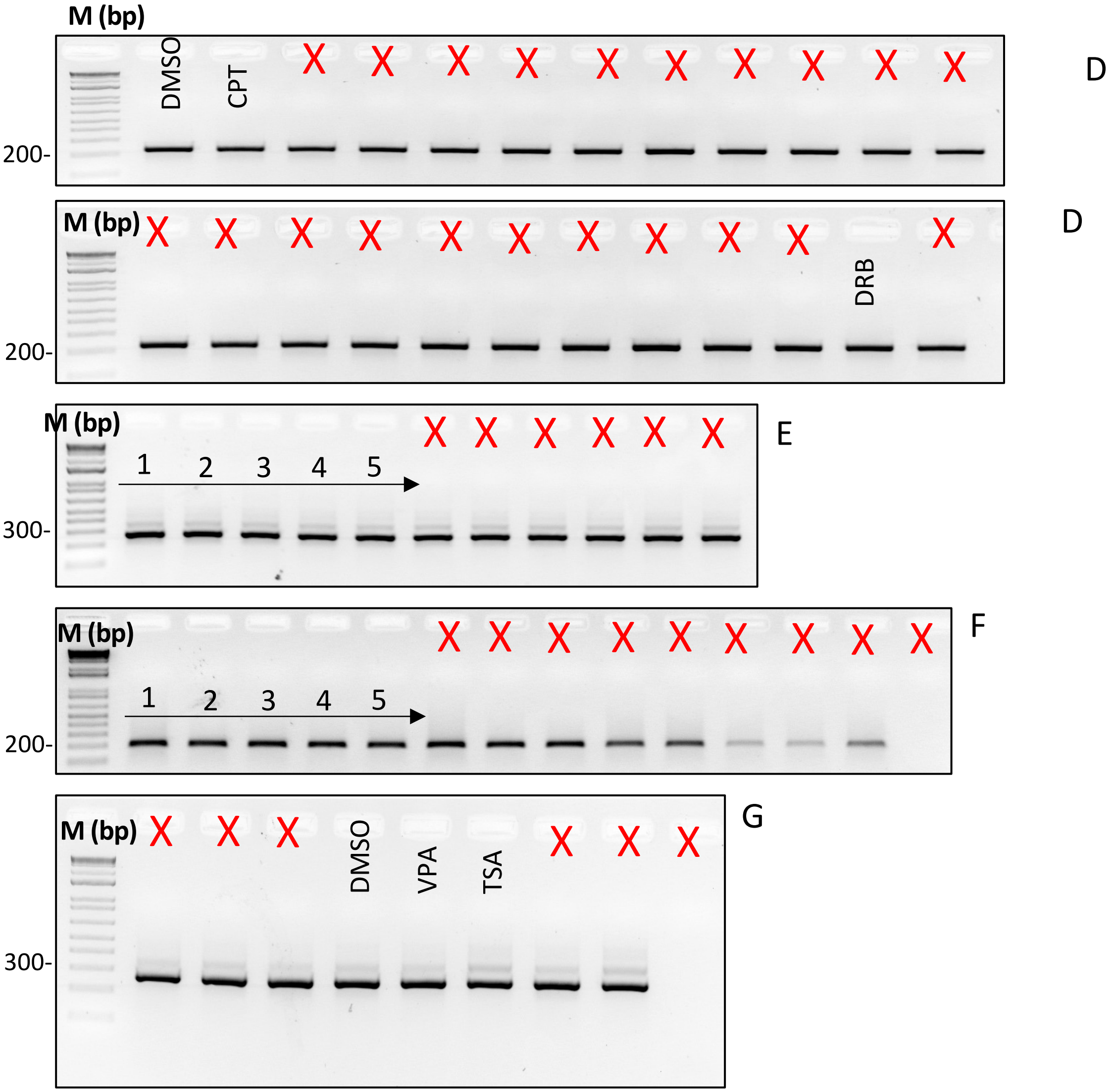
M (bp)
DMSO
CPT
200-
D
M (bp)
DRB
200-
D
M (bp)
1 2 3 4 5
300-
E
M (bp)
1 2 3 4 5
200-
F
M (bp)
DMSO
VPA
TSA
300-
G

Supp Fig 1

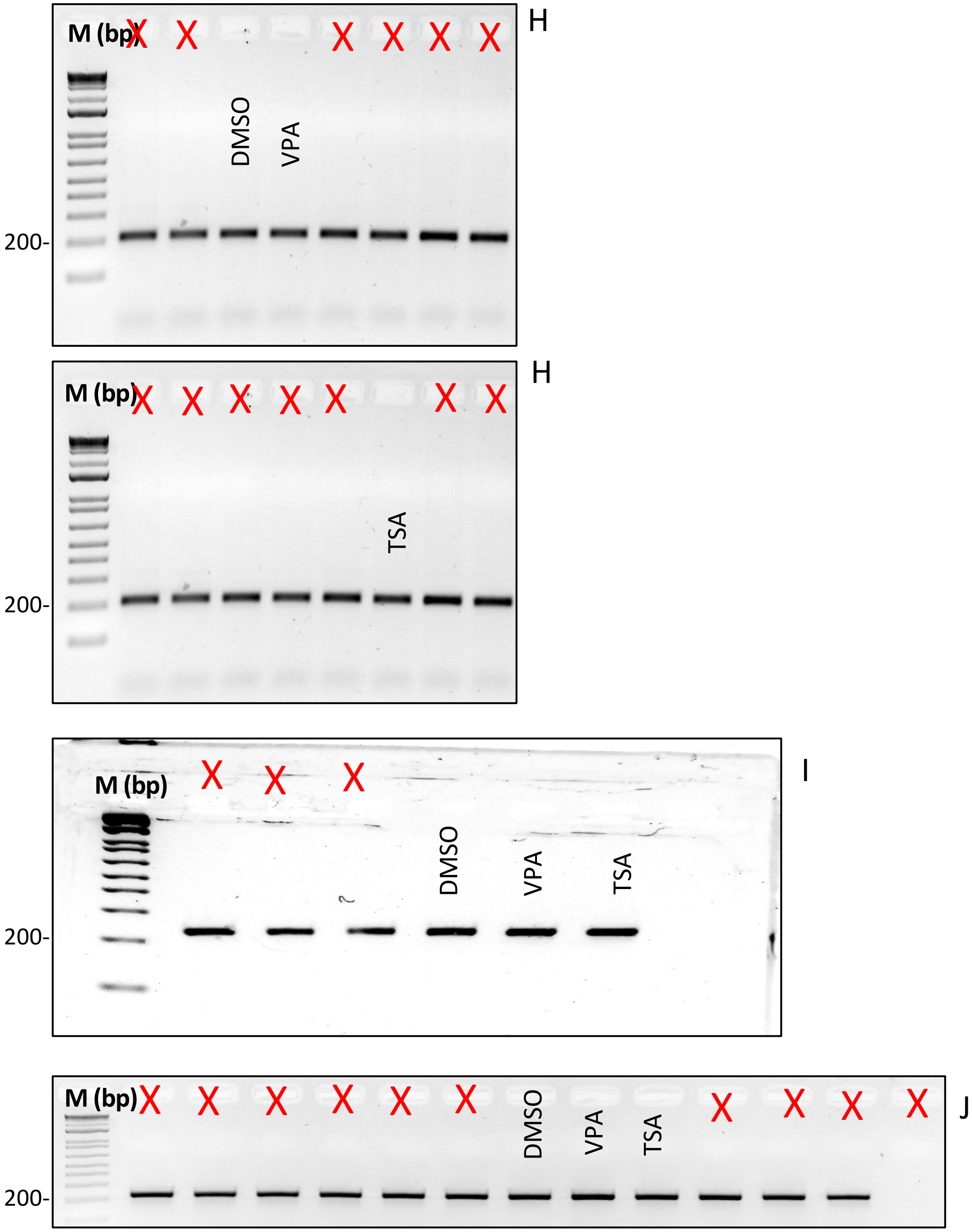
M (bp)
H
DMSO
VPA
200-
M (bp)
H
TSA
200-
M (bp)
I
DMSO
VPA
TSA
200-
M (bp)
J
DMSO
VPA
TSA
200-

Supp Fig 2

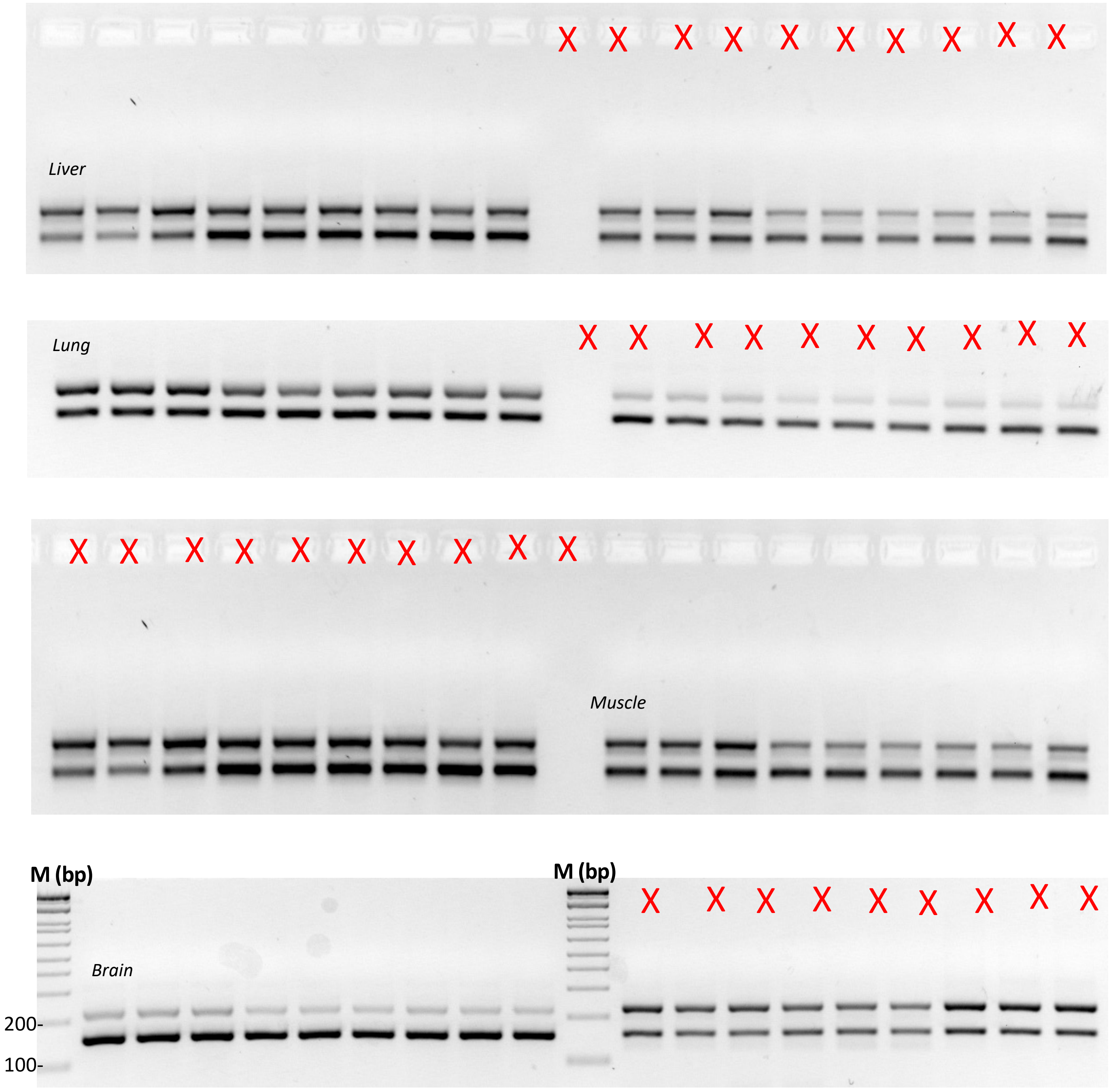

Supp Fig 2

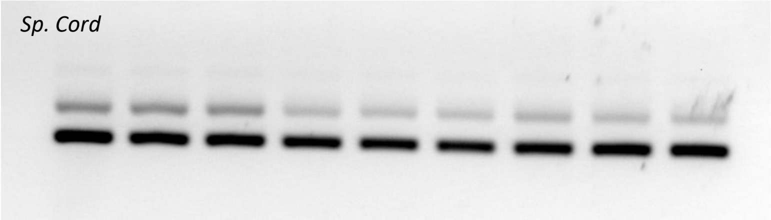

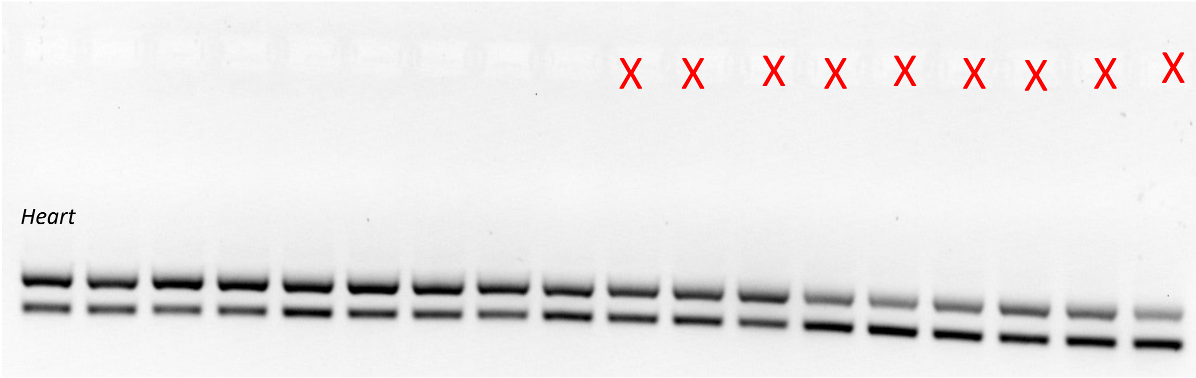

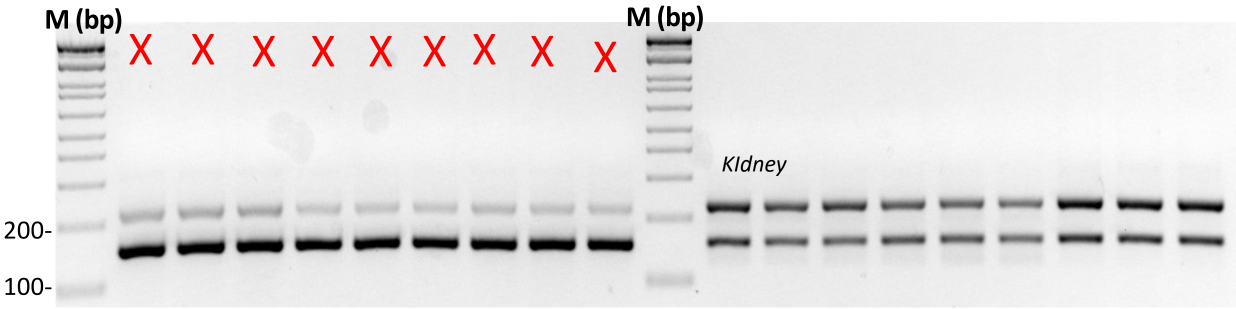

Supp Fig 3

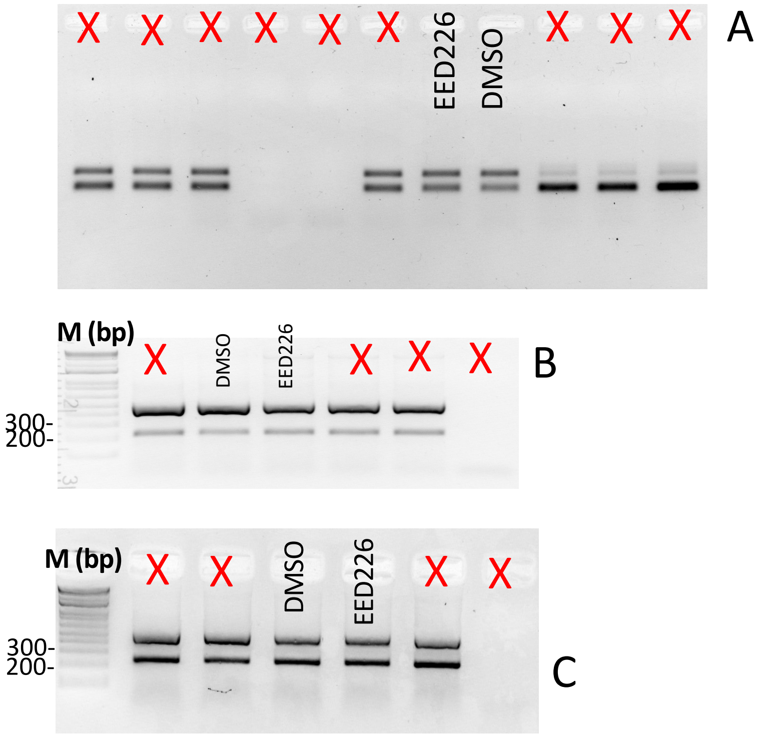

X
X
X
X
X
X
EED226
DMSO
X
X
X
A
M (bp)
X
DMSO
EED226
X
X
X
300-
200-
B
M (bp)
X
X
DMSO
EED226
X
X
300-
200-
C

Supp Fig 3

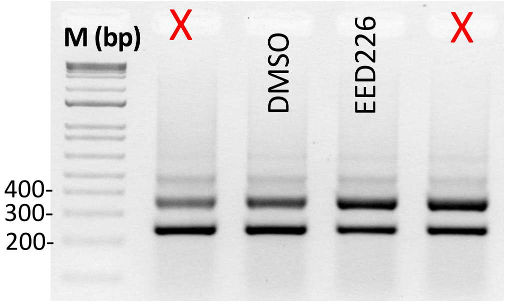

D

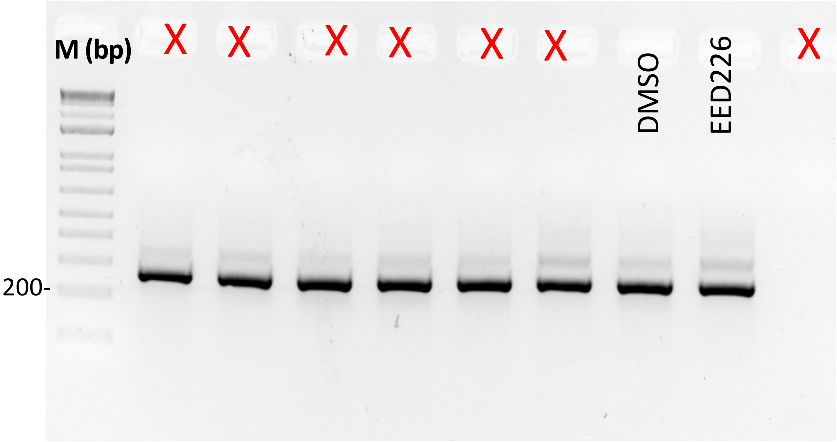

E

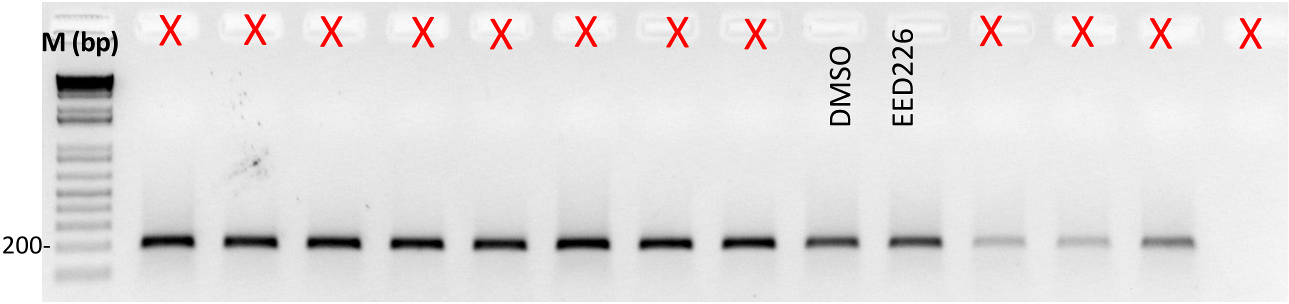

F

Supp Fig 3

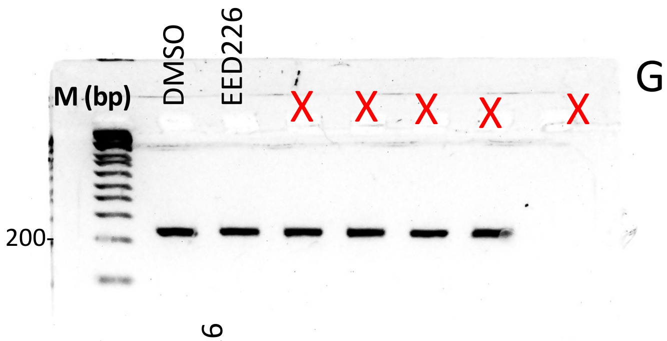

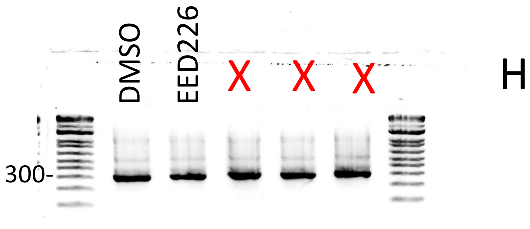

Supp Fig 4

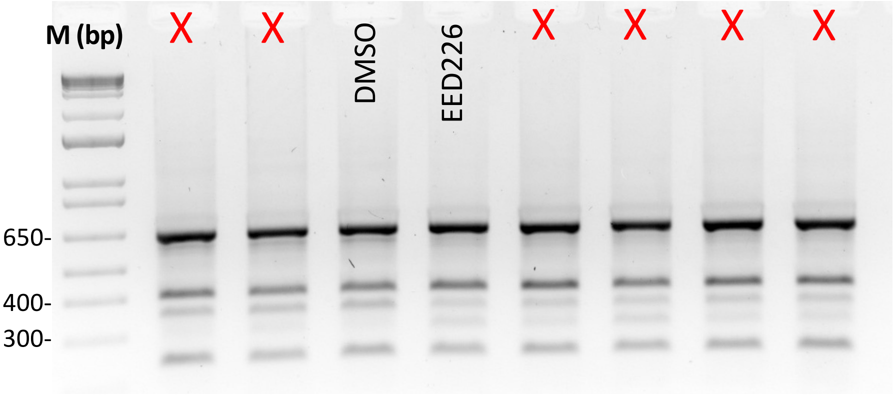

Supplement: S1 File — (PDF) [file pone.0298965.s015.pdf]
